# Carbon dioxide capture and functionalization by bis(*N*-heterocyclic carbene)-borylene complexes

**DOI:** 10.1038/s41467-024-47381-7

**Published:** 2024-04-09

**Authors:** Jun Fan, An-Ping Koh, Chi-Shiun Wu, Ming-Der Su, Cheuk-Wai So

**Affiliations:** 1 School of Chemistry, Chemical Engineering and Biotechnology, Nanyang Technological University, Singapore, 637371 Singapore; 2 Department of Applied Chemistry, National Chiayi University, Chiayi, 60004 Taiwan; 3 Department of Medicinal and Applied Chemistry, Kaohsiung Medical University, Kaohsiung, 80708 Taiwan

**Keywords:** Organometallic chemistry, Synthetic chemistry methodology, Ligands

## Abstract

Derivatives of free monocoordinated borylenes have attracted considerable interest due to their ability to exhibit transition-metal-like reactivity, in particular small molecules capture. However, such complexes are rare as the formation is either endergonic, or the resulting adduct is a transient intermediate that is prone to reaction. Here, we present the synthesis of two bis(*N*-heterocyclic carbene)-borylene complexes capable of capturing and functionalizing carbon dioxide. The capture and subsequent functionalization of CO_2_ by the bis(NHC)-disilylamidoborylene **1** is demonstrated by the formation of the bis(NHC)-isocyanatoborylene-carbon dioxide complex **3**. Reversible capture of CO_2_ is observed using the bis(NHC)-mesitylborylene **2**, and the persistent bis(NHC)-mesitylborylene-carbon dioxide adduct **4** can be stabilized by hydrogen bonding with boric acid. The reactions of **4** with ammonia-borane and aniline demonstrate that the captured CO_2_ can be further functionalized.

## Abstract (web summary)

Bis(*N*-heterocyclic carbene)-borylene complexes are capable of capturing and functionalizing CO_2_, but stable single-site-boron-carbon dioxide adducts are rarely reported. Here, the authors report the synthesis of a stable borylene-CO_2_ complex as well as the functionalization of the captured CO_2_.

## Introduction

The conversion of carbon dioxide (CO_2_) into value-added chemicals has attracted much attention due to the increasing amount of anthropogenic CO_2_ in the atmosphere and consequent climatic problems^1^. Due to the high thermodynamic stability of CO_2_, reactive precious transition metal complexes have been developed to capture, activate, and transform CO_2_ into high-value chemical feedstocks, but some of these elements remain costly and susceptible to potential supply chain issues^2–5^. In this context, the development of sustainable alternatives that possess energetically accessible molecular orbitals to interact with CO_2_ is important.

**Fig. 1:**
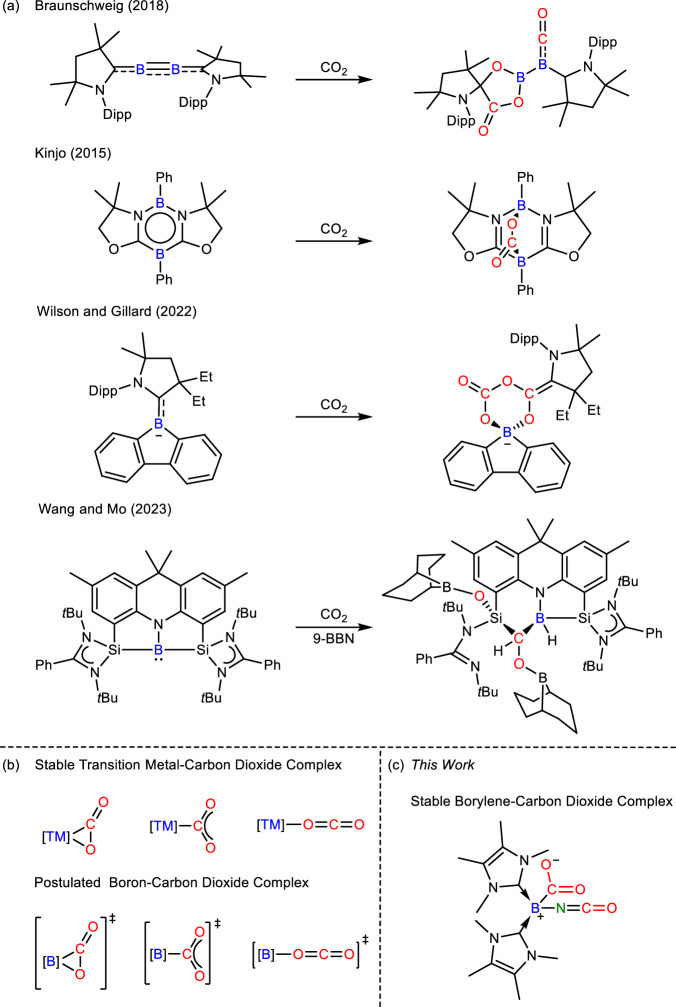
Ambiphilic boron-mediated carbon dioxide activation and its related complexes. **a** Carbon dioxide activation by various ambiphilic boron compounds reported previously. **b** Established structures of stable transition metal-carbon dioxide complexes and postulated analogues based on boron. **c** The first stable borylene-carbon dioxide adduct, bis(NHC)-isocyanatoborylene-carbon dioxide adduct **3**.

Boron derivatives with both strong electrophilic and nucleophilic characters were selected to examine their feasibility in transition-metal-like small-molecules activation. Braunschweig et al. showed that multiply bonded diboron compounds such as bis(NHC)-diborene (NHC = *N*-heterocyclic carbene) and bis(CAAC)-diboracumulene (CAAC = cyclic (alkyl)(amino)carbene) could activate CO_2_ via cycloaddition of B = B and partial B ≡ B bond with CO_2_, respectively (Fig. 1a)^6^. We further illustrated that the B = B double bond in an *N*-phosphinoamidinato NHC-diborene complex was capable of catalyzing hydroboration of CO_2_ with HBpin^7^. Kinjo et al. reported that the detached nucleophilic and electrophilic boron centres in a 6*π*-aromatic 1,3,2,5-diazadiborinine functioned like frustrated Lewis pairs (FLPs) to cooperatively activate CO_2_^8^, which enabled the latter to undergo catalytic *N*-formylation with amines and HBpin (Fig. 1a)^9^. Wilson and Gilliard et al. showed that the CAAC ligand and boron anion in a 9-CAAC-9-borafluorene anion cooperatively activated two equivalents of CO_2_ to form a trioxaborinanone as a perceivable carbon monoxide releasing molecule (Fig. 1a)^10^. Wang and Mo et al. reported that the silylene ligand and borylene center in a distorted T-shaped bis(silylene)amidoborylene cooperatively activated CO_2_ and 9-BBN in hydroboration via a presumed 2-sila-4-boraoxetan-3-one intermediate (Fig. 1a)^11^.

In transition metal-mediated CO_2_ activation, the first step involves simple coordination of CO_2_ with a transition metal in the *η*^2^-CO_2_, *η*^1^-CO_2_-*κC* or *η*^1^-CO_2_-*κO* binding mode (Fig. 1b)^12^. Several stable transition metal complexes of CO_2_ such as Aresta’s [(Cy_3_P)_2_Ni(*η*^2^-CO_2_)]^13^, Herskowitz’s [(diars)_2_M(*η*^1^-CO_2_-*κC*)Cl] (M = Ir, Rh; diars = *o*-phenylenebis(dimethylarsine))^14^ and Gambarotta’s [(ONNO)V(OH)(*η*^1^-CO_2_-*κO*)] (ONNO= [2,4-Me_2_−2-(OH)C_6_H_2_CH_2_]_2_N(CH_2_)_2_NMe_2_)^15^ were isolated and structurally characterized. However, in boron metallomimetic chemistry, single-site-boron complexes of CO_2_ with the composition of B(*η*^2^-CO_2_), B(*η*^1^-CO_2_-*κC*) or B(*η*^1^-CO_2_-*κO*) are unknown. In the activation of CO_2_ by the 9-CAAC-9-borafluorene anion, bis(silylene)amidoborylene or bis(CAAC)-diboracumulene^6,10,11^, Density Functional Theory (DFT) calculations show that the nucleophilic boron center captures CO_2_ in the *η*^1^-CO_2_-κ*C* or *η*^2^-CO_2_ binding mode (Fig. 1b). However, the reaction is either endergonic, or the resulting adduct is a transient intermediate that is prone to react with the electrophilic ligand backbone or the electrophilic boron center. In CO_2_ activation mediated by FLP of phosphine and borane^16^, the phosphine acts as a Lewis base to capture CO_2_ in the *η*^1^-CO_2_-*κC* binding mode while the electrophilic boron center stabilizes the captured CO_2_ via O-coordination^17^. In this context, a stable single-site-boron-carbon dioxide adduct is a highly sought-after compound not only for scientific curiosity, but also for a better understanding of how a single-boron center captures CO_2_ and enables the latter to further react with substrates for functionalization. In this paper, we report the synthesis of a bis(1,3,4,5-tetramethylimidazol-2-ylidene)-bis(trimethylsilyl)amidoborylene and -mesitylborylene and their complexes of CO_2_. The functionalization of the captured CO_2_ is also reported. DFT calculations were performed to elucidate electronic structures.

## Results

**Fig. 2:**
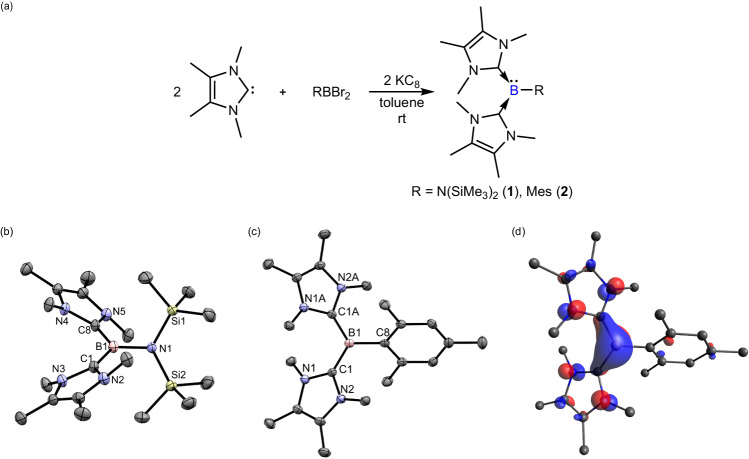
Synthetic routes to compounds 1-2, the molecular structures of 1-2, and the HOMO of 2. **a** Synthetic routes to the bis(NHC)-borylenes **1** and **2**. **b** The molecular structure of **1** obtained by X-ray crystallography. Thermal ellipsoids are shown at 50% probability. All hydrogen atoms are removed for clarity. Selected bond lengths (Å) and angles (deg): B1-C1 1.508(2), B1-C8 1.509(2), B1-N1 1.5327(19), C1-B1-C8 119.28(13), C1-B1-N1 120.63(13), C8-B1-N1 120.09(13). **c** The molecular structure of **2** obtained by X-ray crystallography. Thermal ellipsoids are shown at 50% probability. All hydrogen atoms are removed for clarity. Selected bond lengths (Å) and angles (deg): B1-C1 1.520(3), B1-C1A 1.519(3), B1-C8 1.588(5), C1A-B1-C8 120.99(15), C1-B1-C8 120.99(15), C1-B1-C1A 118.0(3). **d** HOMO (−3.865 eV) of **2** (isovalue 0.06) at M06-2X/def2-TZVP level of theory.

Two equivalents of 1,3,4,5-tetramethylimidazol-2-ylidene (IMe) were reacted with RBBr_2_ [R = N(SiMe_3_)_2_ and mesityl (2,4,6-Me_3_C_6_H_2_)] and KC_8_ in toluene at room temperature to afford the bis(NHC)-disilylamidoborylene [(IMe)_2_B{N(SiMe_3_)_2_}] (**1**, Yield: 73%) and bis(NHC)-mesitylborylene [(IMe)_2_BMes] (**2**, Yield: 42%, Fig. 2a), respectively. They were both isolated as a red crystalline solid from the concentrated reaction mixture. The ^11^B{^1^H} NMR signals of **1** and **2** are 1.6 and −6.8 ppm, respectively. The molecular structures of compounds **1** and **2** obtained by X-ray crystallography show that the boron centers adopt a trigonal planar geometry (Fig. 2b, c). IMe ligands are tilted with respect to the boron centers. The B-C_IMe_ bond lengths in compound **1** (1.508(2)−1.509(2) Å) and **2** (1.519(3)−1.520(3) Å) are almost equal. They are between the B-C_mesityl_ bond (1.588(5) Å) in **2** and B = C double bonds in methylene boranes (1.351–1.488 Å)^18^, indicating some multiple bond character in the B-C_IMe_ bonds due to weak *p*_B_→*p*_C_ *π*-back bonding. In addition, compound **1** has a gauche conformation with respect to the B1-N1 bond. The Si-N-B-C dihedral angle is 58(1)°. The B-N_amido_ bond (1.5327(19) Å) in **1** is typical of a single bond. DFT calculations (M06-2X/Def2-TZVP) of compounds **1** and **2** show that the Highest Occupied Molecular Orbitals (HOMOs) are a dominant *π*-type lone pair orbital on the boron center forming *π*-back bonding with the vacant *p* orbital on IMe, while the Lowest Unoccupied Molecular Orbitals (LUMOs) are the empty *p* orbitals on the carbene centers (Fig. 2d, Supplementary Figs. 33, 34). Their HOMOs are of similar energy level (**1**: −3.801; **2**: −3.865 eV), indicating that their nucleophilicity should be comparable. The Wiberg Bond Index (WBI) shows that the B-C_IMe_ bonds in compounds **1** (WBI: 1.183–1.185) and **2** (WBI: 1.174–1.176) have weak double bond character with reference to the B-C_mesityl_ bond in compound **2** (WBI: 0.949). The WBI of the B-N_amido_ bond in compound **1** is 0.789, which suggests that the B1-N1 bond has a single bond character. The Natural Population Analysis (NPA) charge of the boron center in compound **1** (0.251 e) is higher than that in compound **2** (−0.055 e) due to the inductive effect exerted by the disilylamido substituent. The trend is in line with the ^11^B{^1^H} NMR chemical shift of compounds **1** and **2**.

Synthesis of stable NHC-borylene complexes is a formidable challenge because the weak *π*-accepting property of NHC is insufficient to stabilize the Lewis ambiphilicity of the borylene centers. Robinson and Braunschweig et al. independently showed that NHC-borylene complexes are highly reactive, wherein dimerization or C-H bond activation often occurred^19,20^. In the case of bis(NHC)-borylene complexes where there are two weak *π*-accepting NHC ligands, it is important that the third ligand is strongly *π*-electronic withdrawing for the stabilization of the B lone pair of electrons. Braunschweig et al. reported a bis(NHC)-borylene analogue [(I*i*Pr)_2_BCym] (Cym = (C_5_H_4_)Mn(CO)_3_, I*i*Pr =:C{N(*i*Pr)C(H)}_2_), wherein the lone pair of electrons in the presumed borylene center is stabilized by the B to Mn charge transfer via the Cym ligand leading to a borafulvenium or boratafulvene electronic structure^21^. Driess et al. reported a bis(NHC)-(isocyanide)-borylene cation [(I*i*PrMe)_2_B(CNR)]^+^ (I*i*PrMe =:C{N(*i*Pr)C(Me)}_2_; R = cyclohexyl, tert-butyl), where excess electron density on the boron center delocalizes to the isocyanide ligand to afford a prominent boraketiminium resonance form^22^. As NHCs alone are insufficient to stabilize a borylene, strong *π*-accepting donors such as cyclic (alkyl)(amino)carbenes (CAACs)^23,24^, carbon monoxide or isocyanides^25^ were often used to extensively delocalize the boron lone pair of electrons in other stable bis(Lewis base)-borylene complexes. In contrast, compounds **1** and **2** are rare bis(NHC)-borylene complexes that do not need an extra *π*-electronic withdrawing substituent to stabilize the borylene centers. In addition, the weak *π*-acidity of NHC should preserve the nucleophilic character of borylenes for capturing CO_2_ and subsequently forming stable single-site-boron complexes of CO_2_.

**Fig. 3:**
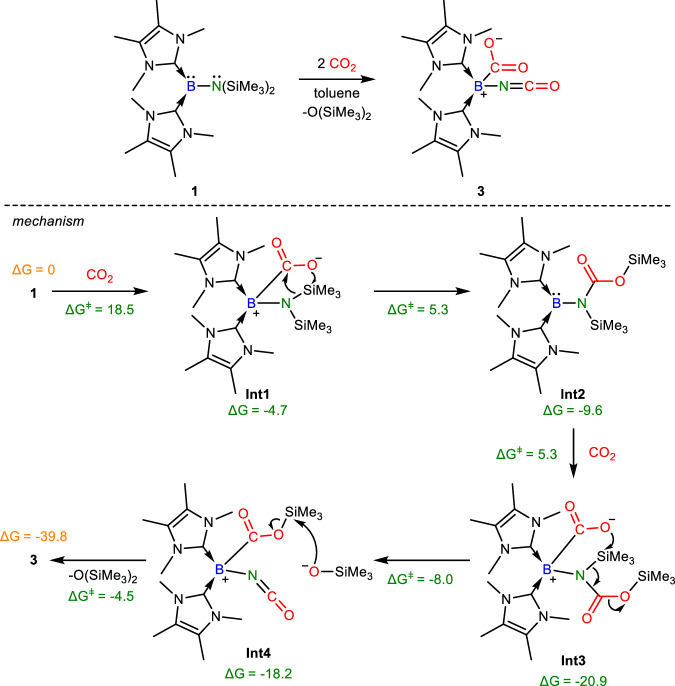
Synthesis of 3 and proposed mechanism for its formation. Reaction of **1** with CO_2_ in toluene at room temperature. (DFT calculations, ΔG and ΔG^ǂ^ in the proposed mechanism are in kcal/mol and were calculated at the M06-2X/def2-TZVP/ IEFPCM(toluene) level of theory).

**Fig. 4:**
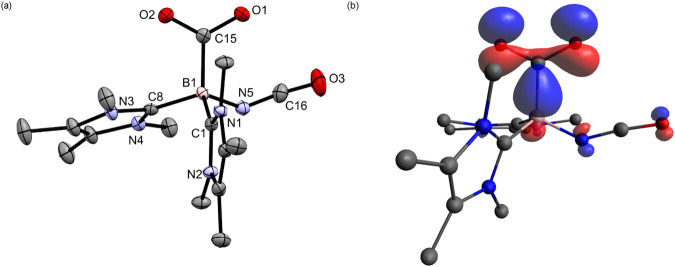
The molecular structure and HOMO of 3. **a** The molecular structure of **3** obtained by X-ray crystallography with thermal ellipsoids shown at 50% probability. All hydrogen atoms are removed for clarity. Selected bond lengths (Å) and angles (deg): B1-C15 1.645(3), C1-B1 1.640(3), C8-B1 1.639(3), B1-N5 1.541(3), C8-B1-C1 106.55(17), C1-B1-N5 105.65(18), N5-B1-C8 111.13(17), O1-C15-O2 123.8(2). **b** HOMO (−6.329 eV) of **3** (isovalue 0.06) at M06-2X/def2-TZVP level of theory, showing the interaction of the lone pair orbital on the B and the π* orbital on CO_2_.

Compound **1** was reacted with CO_2_ (1 bar) in toluene at room temperature, from which the bis(NHC)-isocyanatoborylene-carbon dioxide adduct [(IMe)_2_(OCN)B(*η*^1^-CO_2_-*κ*C)] (**3**, Fig. 3) was isolated as a colorless crystalline solid (Yield: 80%). The ^11^B{^1^H} NMR signal (−16.0 ppm) supports that the boron center is four-coordinate. The molecular structure of **3** obtained by X-ray crystallography (Fig. 4a) shows that the B-C_IMe_ (B1-C1: 1.640(3); B1-C8: 1.639(3) Å) and B-C_CO2_ (1.645(3) Å) bond lengths are typical of single bonds, while the identical C-O bond lengths of the captured CO_2_ (1.263(3) and 1.265(3) Å) indicate the presence of delocalized negative charge. The HOMO of **3** in DFT calculations shows the B-C *σ* orbital formed by the lone pair orbital on the B atom and the π* orbital on CO_2_, leading to a carboxylate anion electronic structure (Fig. 4b). For the formation of **3**, it is proposed that the boron lone pair of electrons attack CO_2_ and the activated CO_2_ moiety in **Int1** inserts into the N-Si bond of the disilylamido substituent to form a carbamate substituent in **Int2**. It further captures CO_2_ to form **Int3**, where the activated CO_2_ attacks the N-Si bond of the carbamate substituent to form the isocyanate and trimethylsilyl carboxylate substituent in **Int4** and Me_3_SiO^-^. The latter reacts with the trimethylsilyl carboxylate substituent in **Int4** to form compound **3** and O(SiMe_3_)_2_. DFT calculations (M06-2X/def2-TZVP/IEFPCM(toluene)) show that the mechanism is feasible.

Compound **3** is the first stable borylene-carbon dioxide adduct^26^. The formation of **3** demonstrates that the boron center in a borylene can directly attack the carbon center of CO_2_, which is a result that has remained unattainable using electrophilic borane. The formation of the isocyanate substituent in compound **3** suggests that functionalization of captured CO_2_ should be feasible. In this context, compound **2** was used to mediate the functionalization of CO_2_ due to the presence of a spectated Mes substituent.

**Fig. 5:**
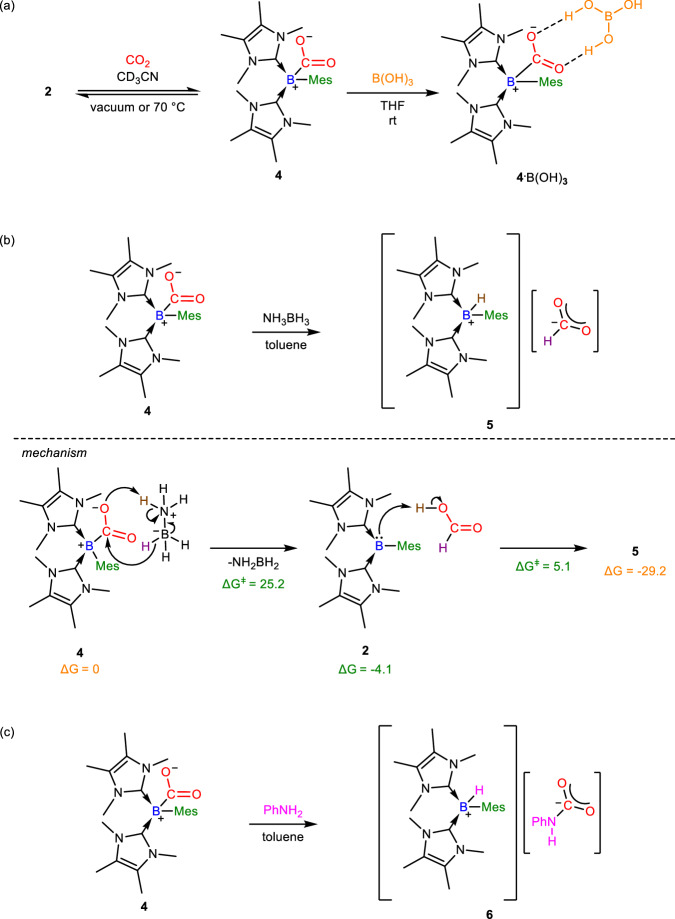
Synthetic routes to compounds 4-6 and the proposed mechanism for the formation of 5. **a** Synthesis of **4** and **4**·B(OH)_3_ from **2**. **b** Reaction of **4** with NH_3_BH_3_ in toluene at room temperature to form **5**. (DFT calculations, ΔG and ΔG^ǂ^ in the proposed mechanism are in kcal/mol and were calculated at the M06-2X/def2-TZVP/ IEFPCM(toluene) level of theory). **c** Reaction of **4** with PhNH_2_ in toluene at room temperature to afford **6**.

The reaction of compound **2** with CO_2_ (1 bar) in CD_3_CN at room temperature afforded the persistent bis(NHC)-mesitylborylene-carbon dioxide adduct [(IMe)_2_(Mes)B(*η*^1^-CO_2_-*κ*C)] (**4,** ^11^B{^1^H} NMR: −15.7 ppm, Fig. 5a). When the reaction mixture was placed under reduced pressure or heated at 70 °C, compound **4** was instantaneously converted back into compound **2** as confirmed by ^11^B{^1^H} NMR spectroscopy, showing that the CO_2_ capture was reversible. Isolating compound **4** by recrystallization was not attained due to its instability.

**Fig. 6:**
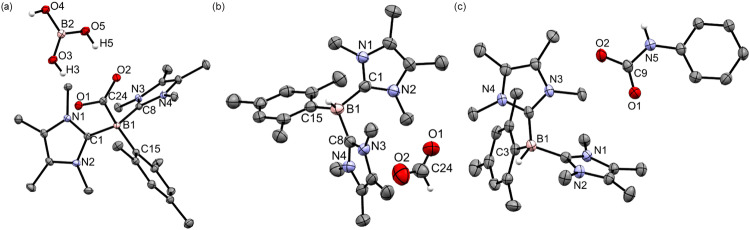
The molecular structures of 4·B(OH)_3_, 5 and 6. **a** The molecular structure of **4**·B(OH)_3_ obtained by X-ray crystallography. Thermal ellipsoids are shown at 50% probability. All hydrogen atoms except for those on boric acid are removed for clarity. Selected bond lengths (Å) and angles (deg): C1-B1 1.6532(16), C8-B1 1.6514(17), C15-B1 1.6608(17), C24-B1 1.6810(17), C24-O1 1.2790(14), C24-O2 1.2643(14), C1-B1-C8 114.24(9), C1-B1-C15 112.85(9), C8-B1-C15 106.30(9), O1-C24-O2 121.60(11), O1-C24-B1 119.45(10), O2-C24-B1 118.68(10). **b** The molecular structure of **5** obtained by X-ray crystallography. Thermal ellipsoids are shown at 50% probability. All hydrogen atoms except for those on the boron and formate are removed for clarity. Selected bond lengths (Å) and angles (deg): C1-B1 1.635(4), C8-B1 1.621(4), C15-B1 1.622(4), C24-O1 1.223(4), C24-O2 1.210(4), O1-C24-O2 133.6(4), C1-B1-C15 117.2(2), C1-B1-C8 108.4(2), C8-B1-C15 116.3(2). **c** The molecular structure of **6** obtained by X-ray crystallography. Thermal ellipsoids are shown at 50% probability. All hydrogen atoms except for those on the boron and carbamate are removed for clarity. Selected bond lengths (Å) and angles (deg): C1-B1 1.618(3), C4-B1 1.631(3), C3-B1 1.643(3), C9-O1 1.246(2), C9-O2 1.268(2), C9-N5 1.406(2), C1-B1-C4 111.65(15), C1-B1-C3 115.09(16), C3-B1-C4 111.65(15), O1-C9-O2 126.14(18), O1-C9-N5 119.53(17), O2-C9-N5 114.33(17).

In carbon monoxide dehydrogenase, CO_2_ is captured by the nucleophilic nickel(0) and electrophilic iron(II) centers, and the activated CO_2_ substrate is further stabilized by hydrogen-bonding with aptly situated amino acid residues^27^. Based on this, an effective single-site catalyst motif, where ligands with pendant proton donors are used to coordinate with a transition metal for the activation of CO_2_ through the stabilization of transition metal-CO_2_ adduct by hydrogen-bonding, has been developed in biomimetic artificial CO_2_ reduction catalysis^2,28^. It is thus anticipated that the captured CO_2_ in compound **4** could be stabilized by hydrogen-bonding by mimicking carbon monoxide dehydrogenase. Boric acid B(OH)_3_ was used to react with compound **4** in THF at room temperature to afford compound **4**·B(OH)_3_ (Fig. 5a), where the captured CO_2_ moiety was stabilized by hydrogen-bonding with B(OH)_3_. Compound **4**·B(OH)_3_ was stable in solution under reduced pressure and was isolated as a colorless crystalline solid (Yield: 53%) from the concentrated reaction mixture. The ^11^B{^1^H} NMR spectrum of compound **4**·B(OH)_3_ shows a signal at −15.8 ppm attributable to the mesityl-bonded boron center, which is comparable with that of compound **4**. The molecular structure of compound **4**·B(OH)_3_ obtained by X-ray crystallography shows that the C-O bonds (1.2643(14), 1.2790(14) Å) are unequal and the oxygen atoms point to two OH substituents of B(OH)_3_ (Fig. 6a). The O···H distances (O2···H5: 1.77 Å; O1···H3: 1.76 Å) indicate the presence of hydrogen bonding. The B-C_IMe_ (B1-C1: 1.6532(16); B1-C8: 1.6514(17) Å) and B-C_CO2_ bonds (1.6810(17) Å) are typical of single bonds. The B-C_mesityl_ bond (1.6608(17) Å) in compound **4·**B(OH)_3_ is significantly lengthened in comparison with that of compound **2**, probably due to the steric congestion at the four-coordinate boron center.

Borylene-mediated hydrogenation of CO_2_ with H_2_ or transfer hydrogenation agents is unknown as yet. As the captured CO_2_ in compound **4·**B(OH)_3_ is capable of interacting with the hydrogen atoms of B(OH)_3_, hydrogenation of the captured CO_2_ should be feasible. As such, NH_3_BH_3_ was used to undergo hydrogenation with compound **4** in toluene at 25 °C to form a formate [(IMe)_2_(Mes)BH](HCO_2_) (**5**, Yield: 51%, Fig. 5b), which was isolated as a colorless crystalline solid from the concentrated reaction mixture. The ^11^B NMR signal of −23.3 ppm (doublet) supports the formation of a B-H bond. The molecular structure of compound **5** obtained by X-ray crystallography shows that the boron center adopts a tetrahedral geometry (Fig. 6b), which is consistent with the upfield ^11^B NMR signal. The OCO skeleton is bent and the C-O bond lengths (1.223(4) and 1.210(4) Å) are shorter than those of **3**, indicating the formation of a formate anion. It is proposed that the hydride from the -BH_3_ moiety of NH_3_BH_3_ attacks the carbon atom of the captured CO_2_, while the negatively charged oxygen atom of the captured CO_2_ in compound **4** abstracts a proton from the -NH_3_ moiety of NH_3_BH_3_ to form formic acid HC(O)OH and regenerate compound **2**. The borylene center in compound **2** then activates the O-H bond of HC(O)OH to form compound **5**. The feasibility of this proposed mechanism is demonstrated by DFT calculations (M06-2X/def2-TZVP/IEFPCM(toluene)). Compound **2** is the first borylene capable of mediating hydrogenation of captured CO_2_ by NH_3_BH_3_ in Lewis-acid-free conditions to form a formate derivative. With the aid of both sterically hindered Lewis acid and base, Stephan et al. showed that hydrogenation of CO_2_ with NH_3_BH_3_ was mediated by an FLP mechanism. In the reaction, the FLP of Mes_3_P and AlX_3_ (X = Cl, Br) activated CO_2_ to form [Mes_3_PC(OAlX_3_)_2_], which subsequently reacted with NH_3_BH_3_ and H_2_O to form methanol via a postulated intermediate [Mes_3_PH][(MeO)_n_AlX_4-n_]^29^. Instead of using NH_3_BH_3_, Dyson and Corminboeuf et al. reported that the FLP of tris(*p*-bromo)tridurylborane (tbtb) and 1,8-diazabicyclo[5.4.0]undec-7-ene (DBU) activated H_2_ under high pressure to form [DBU-H]^+^[H-tbtb]^-^, which reacted with CO_2_ via hydride transfer to afford the formate salt [DBU-H]^+^[HCOO]^-^ and regenerate tbtb^30^. The molecular structure of the formate salt [DBU-H]^+^[HCOO]^-^ is similar to that of compound **5**.

Aromatic amines cannot react with CO_2_ at ambient conditions to form carbamic acids or carbamates^31^. Conversely, the captured CO_2_ in compound **4** could react with PhNH_2_ in toluene at room temperature to form the carbamate [(IMe)_2_(Mes)BH][PhN(H)CO_2_] (**6**, Yield: 72%, Fig. 5c), which was isolated as a colorless crystalline solid from the concentrated reaction mixture. The ^11^B NMR signal of compound **6** is −23.4 ppm (doublet) and its X-ray crystallographic data is consistent with compound **5** (Fig. 6c). The formation of compound **6** illustrates another example of further functionalization of the captured CO_2_ in compound **4**.

## Discussion

This work reports the synthesis of bis(NHC)-disilylamidoborylene **1** and bis(NHC)-mesitylborylene **2** through the reaction of two equivalents of IMe with RBBr_2_ (R = N(SiMe_3_)_2_, 2,4,6-Me_3_C_6_H_2_) and KC_8_. Compounds **1** and **2** are examples of rare bis(NHC)-borylene complexes that do not need an extra *π*-electronic withdrawing substituent to stabilize the borylene centers. The weak *π*-acidity of NHC preserves the nucleophilic character of the borylenes and enables the capture of CO_2_ in the form of the bis(NHC)-isocyanatoborylene-carbon dioxide adduct **3**, which is the first stable single-site-boron complex of CO_2_. Reversible coordination with CO_2_ was also demonstrated using compound **2** to form the persistent bis(NHC)-mesitylborylene-carbon dioxide adduct **4**, which was stabilized by hydrogen bonding with B(OH)_3_ to form compound **4**·B(OH)_3_. Compound **4** was found to be able to undergo hydrogenation with NH_3_BH_3_ to form formate **5** and amination with PhNH_2_ to form carbamate **6**, which demonstrates that the captured CO_2_ can be further functionalized.

## Methods

### General procedures

All operations were carried out under an inert atmosphere of argon gas by standard Schlenk techniques. The synthesis of the starting materials (TMS)_2_NBBr_2_ and MesBBr_2_ were adapted from published procedures, which can be found below. All other chemicals were purchased from Sigma-Aldrich and used directly without further purification. All solvents were dried over K metal or CaH_2_ prior to use. The ^1^H, ^11^B, ^11^B{^1^H}, ^13^C{^1^H}, and ^29^Si{^1^H} NMR spectra were recorded on a JEOL ECA 400 spectrometer or Bruker Avance III 400. The NMR spectra were recorded in deuterated solvents and the chemical shifts are relative to SiMe_4_ for ^1^H, ^13^C and ^29^Si; BF_3_.Et_2_O for ^11^B, respectively. The following abbreviations are used to describe signal multiplicities: s = singlet, d = doublet, m = multiplet, brs = broad singlet. Coupling constants J are given in Hertz (Hz). HRMS spectra were obtained at the Mass Spectrometry Laboratory in the Division of Chemistry and Biological Chemistry, Nanyang Technological University. Melting points were measured with an OptiMelt automated melting point system. Fourier transform infrared (FT-IR) spectra were recorded on a Bruker Alpha FT-IR spectrometer.

**Synthesis of (TMS)**_**2**_**NBBr**_**2**_ adapted from a published procedure^32^.

A hexane solution (2.5 M) of *n*-BuLi (8.0 mL, 20 mmol) was added dropwise into a hexane solution of hexamethyldisilazane (4.19 mL, 20 mmol) at −78 °C. The mixture was allowed to warm to room temperature and stirred for 4 h, then cooled to −78 °C, to which a hexane solution of BBr_3_ (1.90 mL, 20 mmol) was added dropwise. The mixture was gradually warmed to room temperature and stirred overnight. The resulting suspension was filtered, and all volatiles were removed *in vacuo* to give a yellow liquid. Distillation afforded (TMS)_2_NBBr_2_ as a colorless liquid in 32 % yield (2.12 g, 6.65 mmol).

**Synthesis of MesBBr**_**2**_ adapted from a published procedure^33^.

A toluene solution of BBr_3_ (0.95 mL, 10 mmol) was added dropwise into a toluene solution of mesitylcopper(I) (2.01 g, 10 mmol) at −78 °C. The mixture was stirred for 2 h at −78 °C before it was allowed to warm to room temperature and stirred overnight. The resulting suspension was filtered, and all volatiles were removed *in vacuo* to give a yellow liquid. Distillation afforded MesBBr_2_ as a colorless liquid in 72 % yield (2.08 g, 7.17 mmol).

### Synthesis of 1

A toluene solution of (TMS)_2_NBBr_2_ (1.0 mmol) was added into a 100 mL Schlenk flask containing 1,3,4,5-tetramethylimidazolin-2-ylidene (2.0 mmol, 0.25 g) and KC_8_ (2.0 mmol, 0.27 g) at room temperature, following which, the reaction mixture was stirred for 8 h. The resulting bright red-purple suspension was filtered, and the filtrate was concentrated to 10 mL and kept for 3 days at room temperature to afford compound **1** as red block crystals (0.31 g) in 73 % yield. M.p.: 76 °C. ^1^H NMR (399.5 MHz, C_6_D_6_, 25 °C): δ 3.27 (s, 6 H, N-C*H*_3_), 2.41 (s, 6 H, N-C*H*_3_), 1.66 (s, 12 H, C-C*H*_3_), 0.44 (s, 18 H, N(Si(C*H*_3_)_3_)_2_). ^11^B{^1^H} NMR (128 MHz, C_6_D_6_, 25 °C): δ 1.6 (s). ^13^C{^1^H} NMR (101 MHz, C_6_D_6_, 25 °C): δ 121.3, 119.8 (*C* = *C*), 34.9, 34.8 (N*C*H_3_), 10.0, 9.3 (*C*H_3_), 4.7 (N(Si(*C*H_3_)_3_)). ^29^Si{^1^H} NMR (79.4 MHz, C_6_D_6_, 25 °C): δ −1.5 (s). HRMS (ESI): m/z calcd for C_20_H_43_BN_5_Si_2_: 420.3150 [(M + H)]^+^; found: 420.3157.

### Synthesis of 2

A toluene solution of dibromo(2,4,6-trimethylphenyl)borane (MesBBr_2_) (1.0 mmol) was added into a 100 mL Schlenk flask containing 1,3,4,5-tetramethylimidazolin-2-ylidene (2.0 mmol, 0.25 g) and KC_8_ (2.0 mmol, 0.27 g) at room temperature, following which, the reaction mixture was stirred for 8 h. The resulting bright red suspension was filtered, and the filtrate was concentrated to 10 mL and kept for 3 days at room temperature to afford compound **2** as red block crystals (0.16 g) in 42 % yield. M.p.: 94 °C. ^1^H NMR (399.5 MHz, C_6_D_6_, 25 °C): δ 7.20 (s, 2 H, Ar*H*), 2.75 (s, 6 H, N-C*H*_3_), 2.72 (s, 6 H, N-C*H*_3_), 2.57 (s, 6 H, Ar-C*H*_3_), 2.47 (s, 3 H, Ar-C*H*_3_), 1.72 (s, 6 H, C-C*H*_3_), 1.55 (s, 6 H, C-C*H*_3_). ^11^B{^1^H} NMR (128 MHz, C_6_D_6_, 25 °C): δ – 6.8 (s). ^13^C{^1^H} NMR (101 MHz, C_6_D_6_, 25 °C): δ 141.5, 130.9, 127.5 (Ar-*C*), 120.3, 118.8 (*C* = *C*), 34.9, 33.8 (N*C*H_3_), 25.1, 21.7 (Ar-*C*H_3_), 10.0, 9.2 (*C*H_3_). HRMS (ESI): m/z calcd for C_23_H_36_BN_4_: 379.3033 [(M + H)]^+^; found: 379.3035.

### Synthesis of 3

A toluene solution of **1** (0.13 g, 0.3 mmol) in a Schlenk flask was degassed by a freeze–pump–thaw method. Then, CO_2_ (1 bar) was filled. The resulting solution changed from red-purple to colorless immediately. After 30 min of stirring, all volatiles of the resulting suspension were removed under vacuum to give **3** as a colorless solid (0.08 g) in 80% yield. Colorless crystals of **3** were isolated from the saturated acetonitrile solution. M.p.: 81 °C. ^1^H NMR (399.5 MHz, CD_3_CN, 25 °C): δ 3.52 (s, 12 H, N-C*H*_3_), 2.14 (s, 12 H, C-C*H*_3_). ^11^B{^1^H} NMR (128 MHz, CD_3_CN, 25 °C): δ −16.0 (br). ^13^C{^1^H} NMR (101 MHz, CD_3_CN, 25 °C): δ 126.7 (*C* = *C*), 33.2 (N-*C*H_3_), 8.9 (*C*H_3_). HRMS (ESI): m/z calcd for C_16_H_25_BN_5_O_3_: 346.2050 [(M + H)]^+^; found: 346.2056.

### Synthesis of 4

A CD_3_CN solution of **2** (0.04 g, 0.1 mmol) in a J-Young NMR tube was degassed by a freeze–pump–thaw method. Then, CO_2_ (1 bar) was filled. The resulting solution changed from red to colorless immediately. ^1^H NMR (399.5 MHz, CD_3_CN, 25 °C): δ 6.68 (s, 2 H, Ar*H*), 3.29 (s, 12 H, N-C*H*_3_), 2.18 (s, 3 H, Ar-C*H*_3_), 2.13 (s, 12 H, C-C*H*_3_), 1.96 (s, 6 H, Ar-C*H*_3_). ^11^B{^1^H} NMR (128 MHz, CD_3_CN, 25 °C): δ – 15.7 (s). ^13^C{^1^H} NMR (101 MHz, CD_3_CN, 25 °C): δ 144.4, 135.0, 130.2 (Ar-*C*), 126.4 (*C* = *C*), 34.1 (N-*C*H_3_), 24.3, 20.7 (Ar-*C*H_3_), 9.2 (*C*H_3_).

### Synthesis of 4·B(OH)_3_

A THF solution of **2** (0.15 g, 0.4 mmol) in a 100 mL Schlenk flask was degassed by a freeze–pump–thaw method. Then, CO_2_ (1 bar) was filled. The reaction mixture was stirred for 30 min at room temperature. Boric acid B(OH)_3_ (0.03 g, 0.5 mmol) was then added into the colorless solution. After which, the reaction mixture was stirred for 2 h. The resulting suspension was filtered and concentrated to give compound **4**·B(OH)_3_ as colorless crystals (0.11 g) in 53% yield. M.p.: 93 °C. ^1^H NMR (399.5 MHz, CD_3_CN, 25 °C): δ 6.71 (s, 2 H, Ar*H*), 3.25 (s, 12 H, N-C*H*_3_), 2.18 (s, 3 H, Ar-C*H*_3_), 2.14 (s, 12 H, C-C*H*_3_), 1.92 (s, 6 H, Ar-C*H*_3_). ^11^B{^1^H} NMR (128 MHz, CD_3_CN, 25 °C): δ 19.7 (s, *B*(OH)_3_), – 15.8 (s, Ar-*B*). ^13^C{^1^H} NMR (101 MHz, CD_3_CN, 25 °C): δ 144.4, 134.8, 130.2 (Ar-*C*), 126.3 (*C* = *C*), 34.0 (N-*C*H_3_), 24.3, 20.7 (Ar-*C*H_3_), 9.2 (*C*H_3_). HRMS (ESI): m/z calcd for C_24_H_39_B_2_N_4_O_5_: 485.3107 [(M + H)]^+^; found: 485.3121.

### Synthesis of 5

A toluene solution of **2** (0.15 g, 0.4 mmol) in a 100 mL Schlenk flask was degassed by a freeze–pump–thaw method. Then, CO_2_ (1 bar) was filled. The reaction mixture was stirred for 2 h at room temperature. Ammonia borane (NH_3_BH_3_) (0.012 g, 0.4 mmol) was then added into the colorless solution. After which, the reaction mixture was stirred for 4 h. The resulting suspension was filtered and concentrated to give compound **5** as colorless crystals (0.09 g) in 51% yield. M.p.: 262 °C. ^1^H NMR (399.5 MHz, CDCl_3_, 25 °C): δ 6.75 (s, 2 H, Ar*H*), 3.28 (s, 12 H, N-C*H*_3_), 2.23 (s, 3 H, Ar-C*H*_3_), 2.22 (s, 12 H, C-C*H*_3_), 1.77 (s, 6 H, Ar-C*H*_3_). ^11^B NMR (128 MHz, CDCl_3_, 25 °C): δ – 23.3 (d, J = 84.0 Hz). ^13^C{^1^H} NMR (101 MHz, CDCl_3_, 25 °C): δ 167.7 (*C* = O), 141.8, 135.7, 129.3 (Ar-*C*), 126.5 (*C* = *C*), 32.6 (N-*C*H_3_), 23.4, 21.0 (Ar-*C*H_3_), 9.3 (*C*H_3_). HRMS (ESI): m/z calcd for C_24_H_37_BN_4_O_2_: 441.3037 [(M + H)]^+^; found: 441.3038.

### Synthesis of 6

A toluene solution of **2** (0.15 g, 0.4 mmol) in a 100 mL Schlenk flask was degassed by a freeze–pump–thaw method. Then, CO_2_ (1 bar) was filled. The reaction mixture was stirred for 2 h at room temperature. Aniline (PhNH_2_) (0.037 g, 0.4 mmol) was then added into the colorless solution. After which, the reaction mixture was stirred for 4 h. The resulting suspension was filtered and concentrated to give compound **6** as colorless crystals (0.12 g) in 72 % yield. M.p.: 229 °C. ^1^H NMR (399.5 MHz, CDCl_3_, 25 °C): δ 7.37 (d, 1 H, Ar*H*, ^3^J_H-H_ = 7.6 Hz), 7.13-7.09 (m, 1 H, Ar*H*), 7.06-7.02 (m, 1 H, Ar*H*), 6.72 (s, 2 H, Ar*H*), 6.72-6.62 (m, 2 H, Ar*H*), 3.21 (s, 12 H, N-C*H*_3_), 2.19 (s, 3 H, Ar-C*H*_3_), 2.15 (s, 12 H, C-C*H*_3_), 1.72 (s, 6 H, Ar-C*H*_3_). ^11^B NMR (128 MHz, CDCl_3_, 25 °C): δ – 23.4 (d, J = 81.7 Hz). ^13^C{^1^H} NMR (101 MHz, CDCl_3_, 25 °C): δ 162.4 (*C* = O), 158.9, 146.5, 144.3, 141.7, 135.5, 129.3, 129.2, 128.2 (Ar-*C*), 125.9 (*C* = *C*), 118.5, 118.1, 116.6, 115.1(Ar-*C*), 32.4 (N-*C*H_3_), 23.3, 20.9 (Ar-*C*H_3_), 9.1 (*C*H_3_). HRMS (ESI): m/z calcd for C_30_H_42_BN_5_O_2_: 441.3037 [(M + H)]^+^; found: 441.3038.

### Supplementary information

Supplementary Information

Peer Review File

### Source data

Source Data

## Data Availability

All data generated or analyzed during this study are included in this manuscript (and its Supplementary Information). Details about materials and methods, experimental procedures, characterization data, and NMR spectra are available in the Supplementary Information. The optimized cartesian coordinates are provided in the Source Data file. The structures of **1**–**6** in the solid state were determined by single-crystal X-ray diffraction studies and the crystallographic data for these structures have been deposited at the Cambridge Crystallographic Data Centre (CCDC) under deposition numbers 2235472 (**1**), 2257718 (**2**), 2235466 (**3**), 2257719 (**4**^.^B(OH)_3_), 2307337 (**5**), 2307338 (**6**). These data can be obtained free of charge from via www.ccdc.cam.ac.uk/data_request/cif. All data are also available from corresponding authors upon request. Source data are provided with this paper.
