# Supplementary material for: Carbon dioxide capture and functionalization by bis(N-heterocyclic carbene)-borylene complexes
Source: Nat Commun. 2024 Apr 9;15:3052. doi: 10.1038/s41467-024-47381-7 (PMC11003992; doi:10.1038/s41467-024-47381-7)
Supplement: Supplementary file 1 — Supplementary Information [file 41467_2024_47381_MOESM1_ESM.pdf]

Supplementary Information for

Carbon Dioxide Capture and Functionalization by Bis(*N*-Heterocyclic Carbene)-Borylene Complexes

Jun Fan[1], An-Ping Koh[1], Chi-Shiun Wu[2], Ming-Der Su[2,3*] and Cheuk-Wai So[1*]

[1] *School of Chemistry, Chemical Engineering and Biotechnology, Nanyang Technological University, 637371 Singapore.*

[2]*Department of Applied Chemistry, National Chiayi University, Chiayi 60004, Taiwan.*

[3]*Department of Medicinal and Applied Chemistry, Kaohsiung Medical University, Kaohsiung 80708, Taiwan.*

Contents

1. Supplementary Methods

Experimental Procedures

NMR Spectra

IR Spectra

2. Supplementary Discussion

X-Ray Crystallography Data

Computational Studies

3. Supplementary References

1. Supplementary Methods

Experimental Procedures

General procedures

All operations were carried out under an inert atmosphere of argon gas by standard Schlenk techniques. The synthesis of the starting materials $(TMS)_2NBBr_2$ and $MesBBr_2$ were adapted from published procedures, which can be found below. All other chemicals were purchased from Sigma-Aldrich and used directly without further purification. All solvents were dried over K metal or CaH_2 prior to use. The ^{1}H, ^{11}B, $^{11}B\{^{1}H\}$, $^{13}C\{^{1}H\}$, and $^{29}Si\{^{1}H\}$ NMR spectra were recorded on a JEOL ECA 400 spectrometer or Bruker Avance III 400. The NMR spectra were recorded in deuterated solvents and the chemical shifts are relative to $SiMe_4$ for ^{1}H, ^{13}C and ^{29}Si; $BF_3.Et_2O$ for ^{11}B, respectively. The following abbreviations are used to describe signal multiplicities: s = singlet, d = doublet, m = multiplet, brs = broad singlet. Coupling constants J are given in Hertz (Hz). HRMS spectra were obtained at the Mass Spectrometry Laboratory in the Division of Chemistry and Biological Chemistry, Nanyang Technological University. Melting points were measured with an OptiMelt automated melting point system. Fourier transform infrared (FT-IR) spectra were recorded on a Bruker Alpha FT-IR spectrometer.

Synthesis of $(TMS)_2NBBr_2$ adapted from a published procedure.[1] A hexane solution (2.5 M) of *n*-BuLi (8.0 mL, 20 mmol) was added dropwise into a hexane solution of hexamethyldisilazane (4.19 mL, 20 mmol) at -78 °C. The mixture was allowed to warm to room temperature and stirred for 4 h, then cooled to -78 °C, to which a hexane solution of BBr_3 (1.90 mL, 20 mmol) was added dropwise. The mixture was gradually warmed to room temperature and stirred overnight. The resulting suspension was filtered, and all volatiles were removed *in vacuo* to give a yellow liquid. Distillation afforded $(TMS)_2NBBr_2$ as a colorless liquid in 32 % yield (2.12 g, 6.65 mmol).

Synthesis of $MesBBr_2$ adapted from a published procedure.[2] A toluene solution of BBr_3 (0.95 mL, 10 mmol) was added dropwise into a toluene solution of mesitylcopper(I) (2.01 g, 10 mmol) at -78 °C. The mixture was stirred for 2 h at -78 °C before it was allowed to warm to room temperature and stirred overnight. The resulting suspension was filtered, and all volatiles were removed *in vacuo* to give a yellow liquid. Distillation afforded $MesBBr_2$ as a colorless liquid in 72 % yield (2.08 g, 7.17 mmol).

Synthesis of 1

A toluene solution of $(TMS)_2NBBr_2$ (1.0 mmol) was added into a 100 mL Schlenk flask containing 1,3,4,5-tetramethylimidazolin-2-ylidene (2.0 mmol, 0.25 g) and KC_8 (2.0 mmol, 0.27 g) at room temperature, following which, the reaction mixture was stirred for 8 h. The resulting bright red purple suspension was filtered, and the filtrate was concentrated to 10 mL and kept for 3 days at room temperature to afford compound **1** as red block crystals (0.31 g) in 73 % yield. M.p.: 76 °C. ^{1}H NMR (399.5 MHz, C_6D_6, 25 °C): δ 3.27 (s, 6 H, N-CH_3), 2.41 (s, 6 H, N-CH_3), 1.66 (s, 12 H, C-CH_3), 0.44 (s, 18 H, N(Si(CH_3)$_3$)$_2$). $^{11}B\{^{1}H\}$ NMR (128 MHz, C_6D_6, 25 °C): δ 1.6 (s). $^{13}C\{^{1}H\}$ NMR (101 MHz, C_6D_6, 25 °C): δ 121.3, 119.8 (*C*=*C*), 34.9, 34.8 (N*C*H_3), 10.0, 9.3 (*C*H_3), 4.7 (N(Si(*C*H_3)$_3$)). $^{29}Si\{^{1}H\}$ NMR (79.4 MHz, C_6D_6, 25 °C): δ -1.5 (s). HRMS (ESI): m/z calcd for $C_{20}H_{43}BN_5Si_2$: 420.3150 [(M + H)]+; found: 420.3157.

Synthesis of 2

A toluene solution of dibromo(2,4,6-trimethylphenyl)borane ($MesBBr_2$) (1.0 mmol) was added into a 100 mL Schlenk flask containing 1,3,4,5-tetramethylimidazolin-2-ylidene (2.0 mmol, 0.25 g) and KC_8 (2.0 mmol, 0.27 g) at room temperature, following which, the reaction mixture was stirred for 8 h. The resulting bright red suspension was filtered, and the filtrate was concentrated to 10 mL and kept for 3 days at room temperature to afford compound **2** as red block crystals (0.16 g) in 42 % yield. M.p.: 94 °C. ^1H NMR (399.5 MHz, C_6D_6, 25 °C): δ 7.20 (s, 2 H, Ar*H*), 2.75 (s, 6 H, N-C*H*3), 2.72 (s, 6 H, N-C*H*3), 2.57 (s, 6 H, Ar-C*H*3), 2.47 (s, 3 H, Ar-C*H*3), 1.72 (s, 6 H, C-C*H*3), 1.55 (s, 6 H, C-C*H*3). $^{11}B\{^1H\}$ NMR (128 MHz, C_6D_6, 25 °C): δ – 6.8 (s). $^{13}C\{^1H\}$ NMR (101 MHz, C_6D_6, 25 °C): δ 141.5, 130.9, 127.5 (Ar-*C*), 120.3, 118.8 (*C*=*C*), 34.9, 33.8 (N*C*H3), 25.1, 21.7 (Ar-*C*H3), 10.0, 9.2 (*C*H3). HRMS (ESI): m/z calcd for $C_{23}H_{36}BN_4$: 379.3033 [(M + H)]+; found: 379.3035.

Synthesis of 3

A toluene solution of **1** (0.13 g, 0.3 mmol) in a Schlenk flask was degassed by a freeze–pump–thaw method. Then, CO_2 (1 bar) was filled. The resulting solution changed from red purple to colorless immediately. After 30 min of stirring, all volatiles of the resulting suspension were removed under vacuum to give **3** as a colorless solid (0.08 g) in 80% yield. Colorless crystals of **3** were isolated from the saturated acetonitrile solution. M.p.: 81 °C. ^1H NMR (399.5 MHz, CD_3CN, 25 °C): δ 3.52 (s, 12 H, N-C*H*3), 2.14 (s, 12 H, C-C*H*3). $^{11}B\{^1H\}$ NMR (128 MHz, CD_3CN, 25 °C): δ -16.0 (br). $^{13}C\{^1H\}$ NMR (101 MHz, CD_3CN, 25 °C): δ 126.7 (*C*=*C*), 33.2 (N-*C*H3), 8.9 (*C*H3). HRMS (ESI): m/z calcd for $C_{16}H_{25}BN_5O_3$: 346.2050 [(M + H)]+; found: 346.2056.

Synthesis of 4

A CD_3CN solution of **2** (0.04 g, 0.1 mmol) in a J-Young NMR tube was degassed by a freeze–pump–thaw method. Then, CO_2 (1 bar) was filled. The resulting solution changed from red to colorless immediately. ^1H NMR (399.5 MHz, CD_3CN, 25 °C): δ 6.68 (s, 2 H, Ar*H*), 3.29 (s, 12 H, N-C*H*3), 2.18 (s, 3 H, Ar-C*H*3), 2.13 (s, 12 H, C-C*H*3), 1.96 (s, 6 H, Ar-C*H*3). $^{11}B\{^1H\}$ NMR (128 MHz, CD_3CN, 25 °C): δ – 15.7 (s). $^{13}C\{^1H\}$ NMR (101 MHz, CD_3CN, 25 °C): δ 144.4, 135.0, 130.2 (Ar-*C*), 126.4 (*C*=*C*), 34.1 (N-*C*H3), 24.3, 20.7 (Ar-*C*H3), 9.2 (*C*H3).

Synthesis of 4·B(OH)3

A THF solution of **2** (0.15 g, 0.4 mmol) in a 100 mL Schlenk flask was degassed by a freeze–pump–thaw method. Then, CO_2 (1 bar) was filled. The reaction mixture was stirred for 30 min at room temperature. Boric acid $B(OH)_3$ (0.03 g, 0.5 mmol) was then added into the colorless solution. After which, the reaction mixture was stirred for 2 h. The resulting suspension was filtered and concentrated to give compound **4**·$B(OH)_3$ as colorless crystals (0.11 g) in 53% yield. M.p.: 93 °C. ^1H NMR (399.5 MHz, CD_3CN, 25 °C): δ 6.71 (s, 2 H, Ar*H*), 3.25 (s, 12 H, N-C*H*3), 2.18 (s, 3 H, Ar-C*H*3), 2.14 (s, 12 H, C-C*H*3), 1.92 (s, 6 H, Ar-C*H*3). $^{11}B\{^1H\}$ NMR (128 MHz, CD_3CN, 25 °C): δ 19.7 (s, *B*(OH)3), – 15.8 (s, Ar-*B*). $^{13}C\{^1H\}$ NMR (101 MHz, CD_3CN, 25 °C): δ 144.4, 134.8, 130.2 (Ar-*C*), 126.3 (*C*=*C*), 34.0 (N-*C*H3), 24.3, 20.7 (Ar-*C*H3), 9.2 (*C*H3). HRMS (ESI): m/z calcd for $C_{24}H_{39}B_2N_4O_5$: 485.3107 [(M + H)]+; found: 485.3121.

Synthesis of 5

A toluene solution of **2** (0.15 g, 0.4 mmol) in a 100 mL Schlenk flask was degassed by a freeze–pump–thaw method. Then, CO_2 (1 bar) was filled. The reaction mixture was stirred for 2 h at room temperature. Ammonia borane (NH_3BH_3) (0.012 g, 0.4 mmol) was then added into the colorless solution. After which, the reaction mixture was stirred for 4 h. The resulting suspension was filtered and concentrated to give compound **5** as colorless crystals (0.09 g) in 51% yield. M.p.: 262 °C. ^{1}H NMR (399.5 MHz, $CDCl_3$, 25 °C): δ 6.75 (s, 2 H, Ar*H*), 3.28 (s, 12 H, N-C*H*$_3$), 2.23 (s, 3 H, Ar-C*H*$_3$), 2.22 (s, 12 H, C-C*H*$_3$), 1.77 (s, 6 H, Ar-C*H*$_3$). ^{11}B NMR (128 MHz, $CDCl_3$, 25 °C): δ – 23.3 (d, J = 84.0 Hz). ^{13}C{^{1}H} NMR (101 MHz, $CDCl_3$, 25 °C): δ 167.7 (*C*=O), 141.8, 135.7, 129.3 (Ar-*C*), 126.5 (*C*=*C*), 32.6 (N-*C*H$_3$), 23.4, 21.0 (Ar-*C*H$_3$), 9.3 (*C*H$_3$). HRMS (ESI): m/z calcd for $C_{24}H_{37}BN_4O_2$: 441.3037 [(M + H)]+; found: 441.3038.

Synthesis of 6

A toluene solution of **2** (0.15 g, 0.4 mmol) in a 100 mL Schlenk flask was degassed by a freeze–pump–thaw method. Then, CO_2 (1 bar) was filled. The reaction mixture was stirred for 2 h at room temperature. Aniline ($PhNH_2$) (0.037 g, 0.4 mmol) was then added into the colorless solution. After which, the reaction mixture was stirred for 4 h. The resulting suspension was filtered and concentrated to give compound **6** as colorless crystals (0.12 g) in 72 % yield. M.p.: 229 °C. ^{1}H NMR (399.5 MHz, $CDCl_3$, 25 °C): δ 7.37 (d, 1 H, Ar*H*, $^3J_{H\text{-}H}$ = 7.6 Hz), 7.13-7.09 (m, 1 H, Ar*H*), 7.06-7.02 (m, 1 H, Ar*H*), 6.72 (s, 2 H, Ar*H*), 6.72-6.62 (m, 2 H, Ar*H*), 3.21 (s, 12 H, N-C*H*$_3$), 2.19 (s, 3 H, Ar-C*H*$_3$), 2.15 (s, 12 H, C-C*H*$_3$), 1.72 (s, 6 H, Ar-C*H*$_3$). ^{11}B NMR (128 MHz, $CDCl_3$, 25 °C): δ – 23.4 (d, J = 81.7 Hz). ^{13}C{^{1}H} NMR (101 MHz, $CDCl_3$, 25 °C): δ 162.4 (*C*=O), 158.9, 146.5, 144.3, 141.7, 135.5, 129.3, 129.2, 128.2(Ar-*C*), 125.9 (*C*=*C*), 118.5, 118.1, 116.6, 115.1(Ar-*C*), 32.4 (N-*C*H$_3$), 23.3, 20.9 (Ar-*C*H$_3$), 9.1 (*C*H$_3$). HRMS (ESI): m/z calcd for $C_{30}H_{42}BN_5O_2$: 441.3037 [(M + H)]+; found: 441.3038.

NMR spectra of isolated compounds

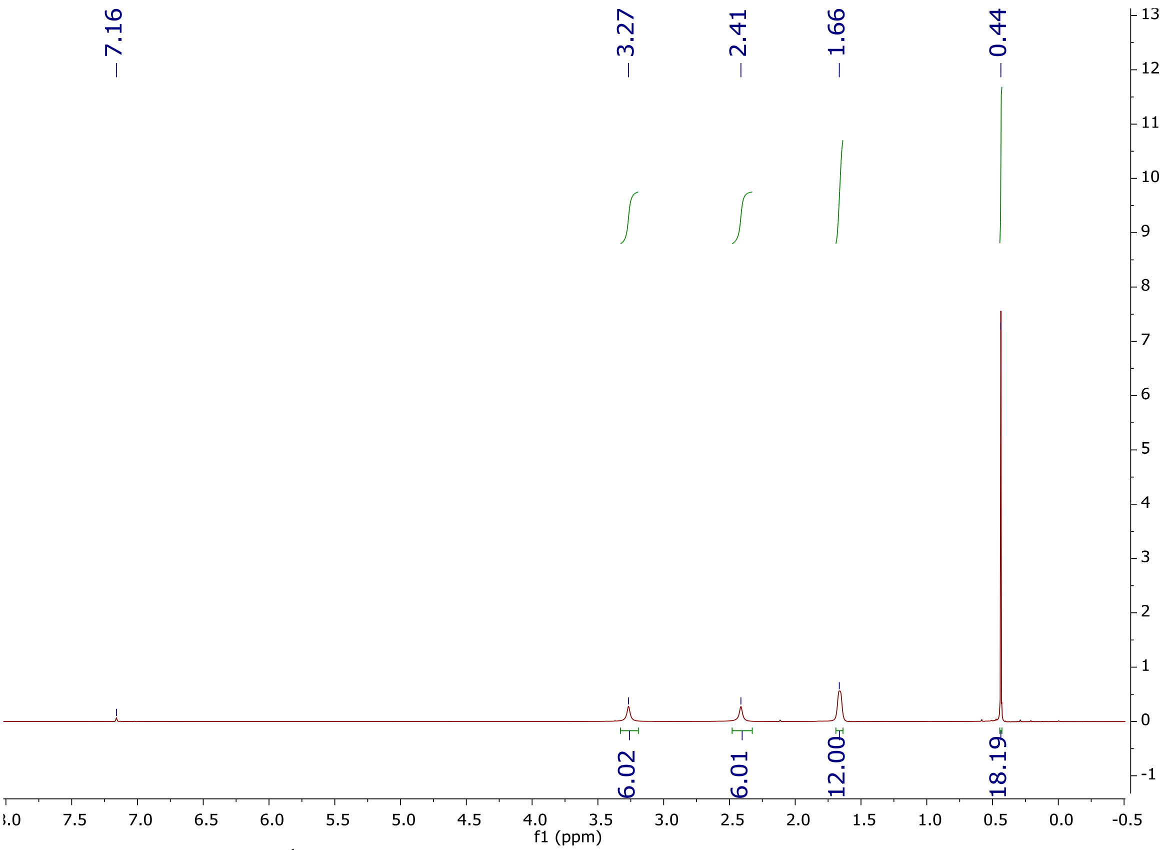

Supplementary Fig. 1. ^{1}H NMR spectrum of **1** (in C_6D_6).

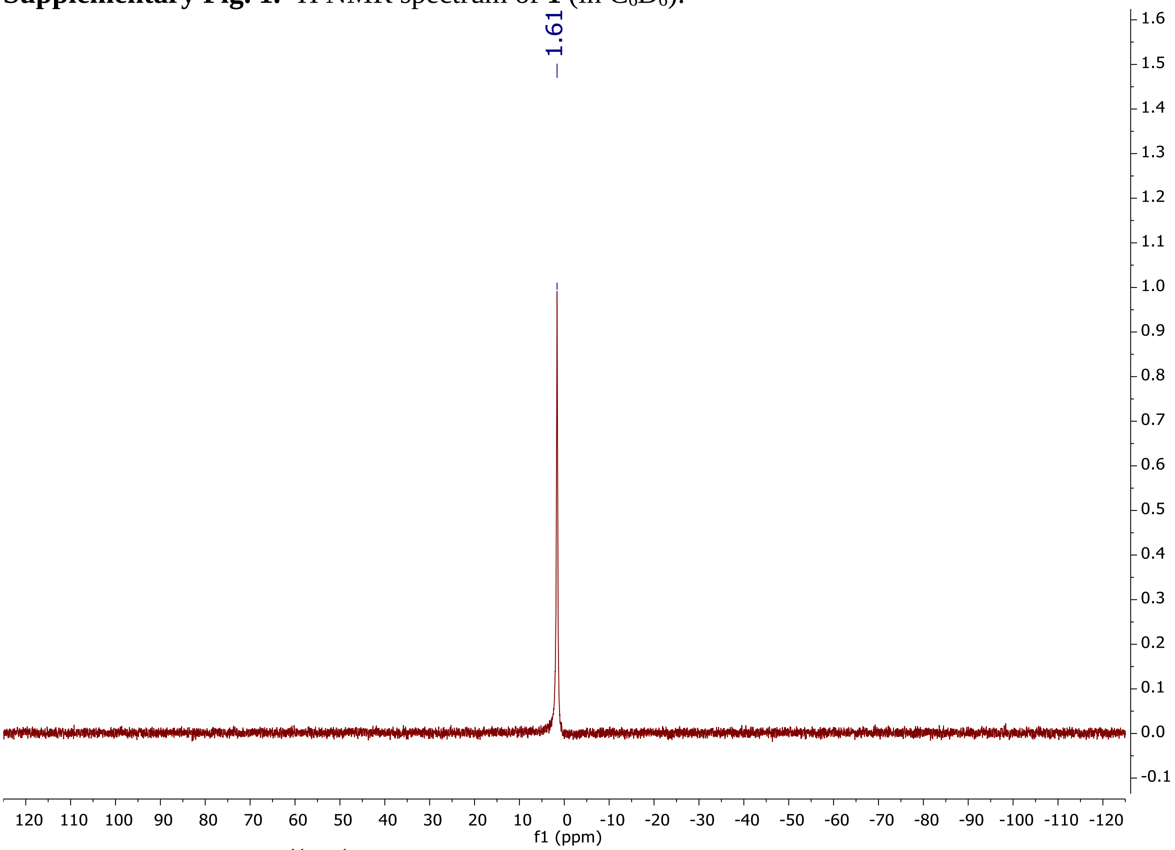

Supplementary Fig. 2. $^{11}B\{^{1}H\}$ NMR spectrum of **1** (in C_6D_6).

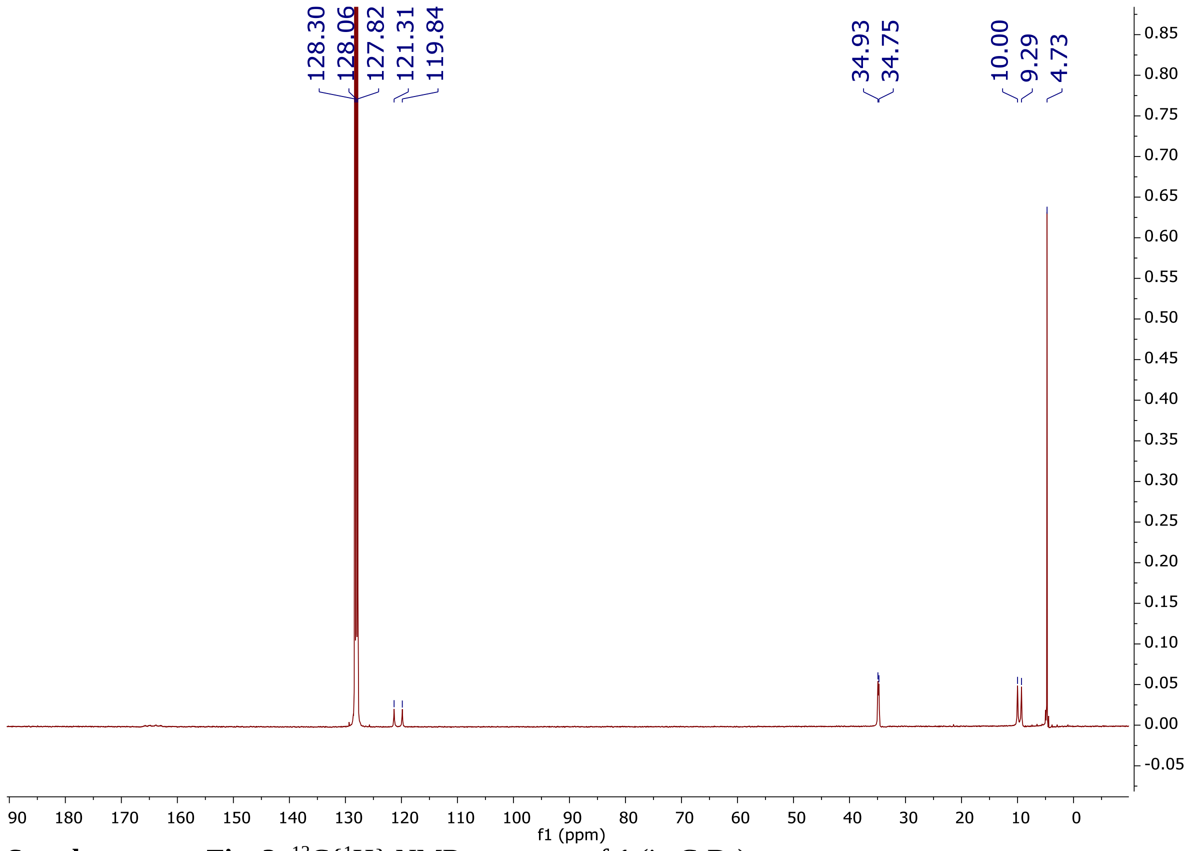

Supplementary Fig. 3. $^{13}C\{^{1}H\}$ NMR spectrum of **1** (in C_6D_6).

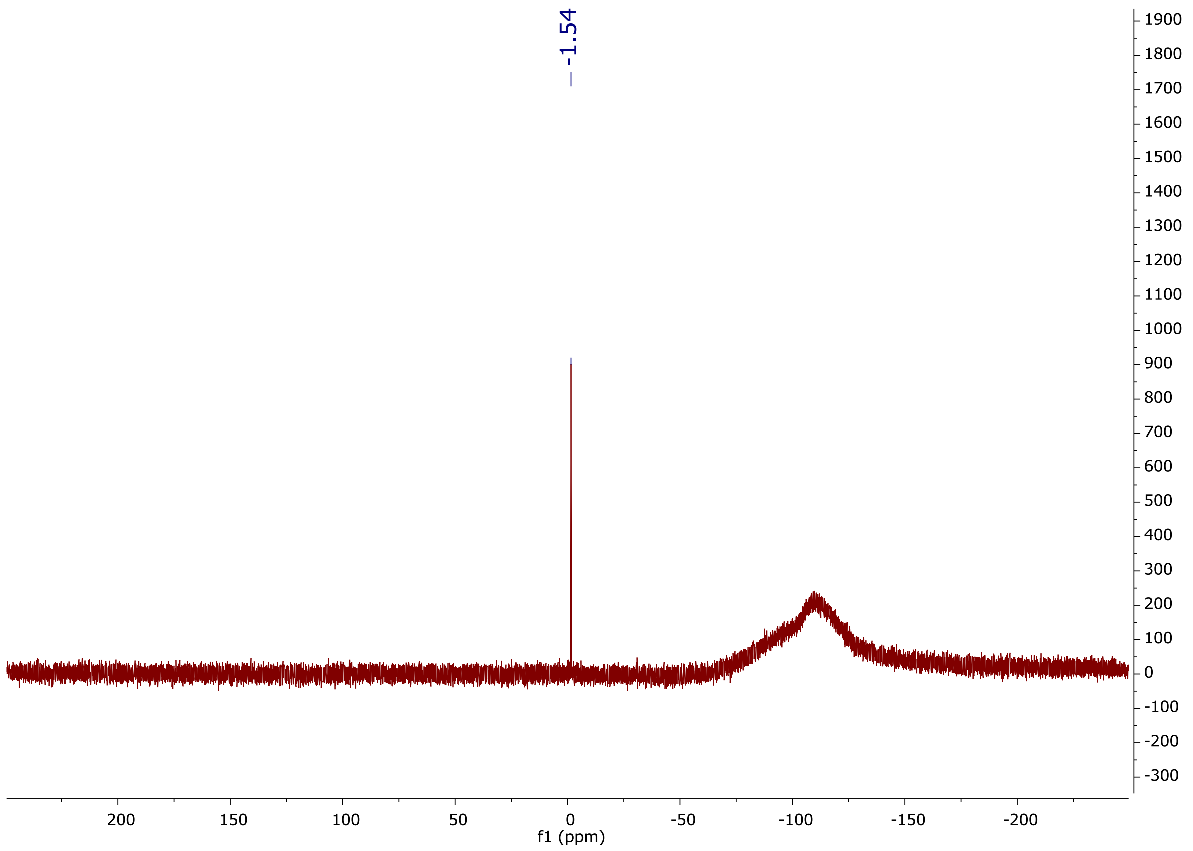

Supplementary Fig. 4. $^{29}Si\{^{1}H\}$ NMR spectrum of **1** (in C_6D_6).

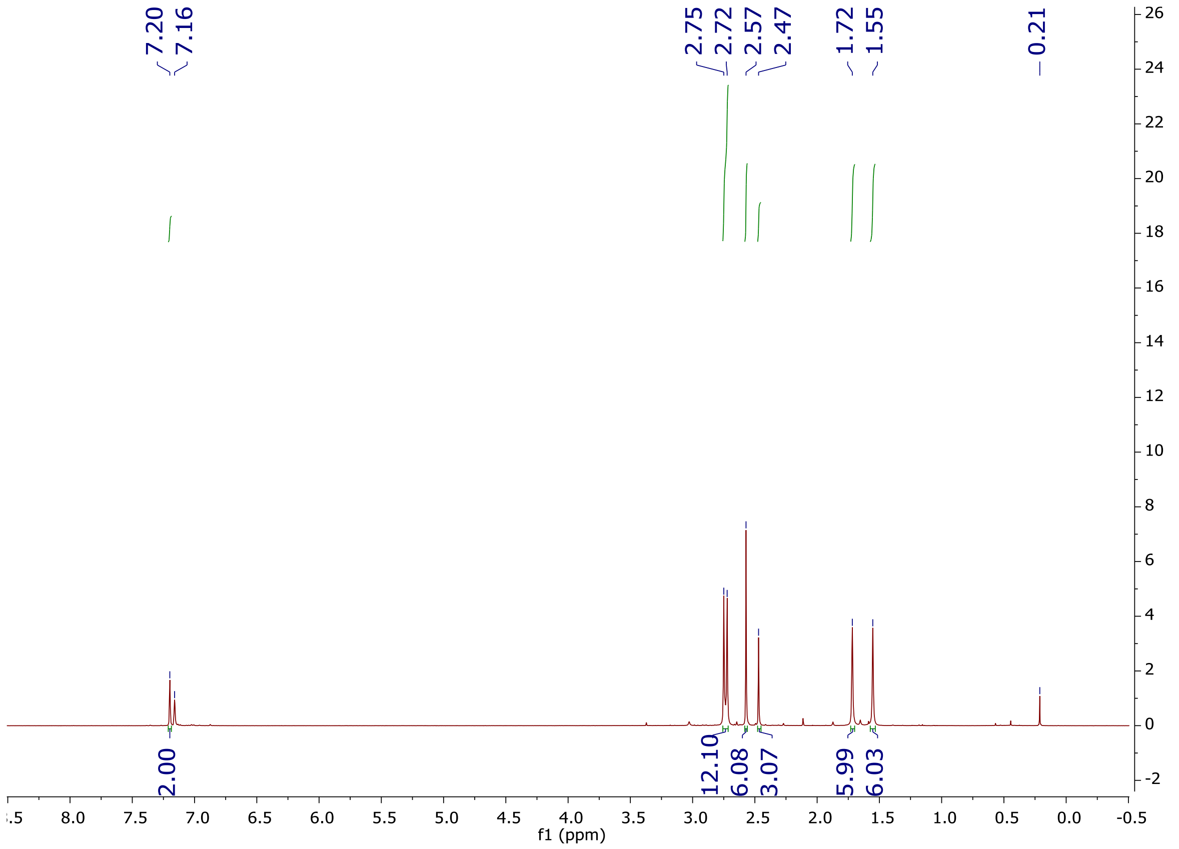

Supplementary Fig. 5. ^{1}H NMR spectrum of **2** (in C_6D_6).

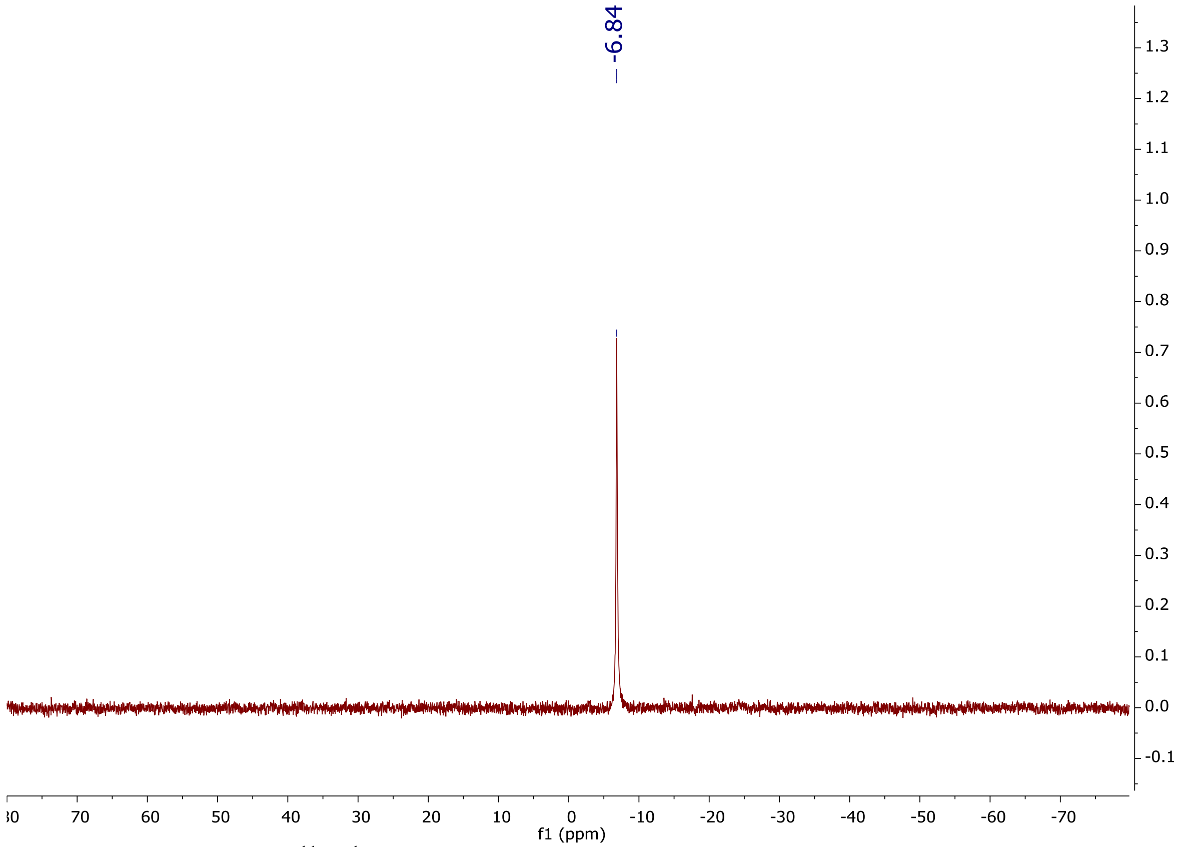

Supplementary Fig. 6. $^{11}B\{^{1}H\}$ NMR spectrum of **2** (in C_6D_6)

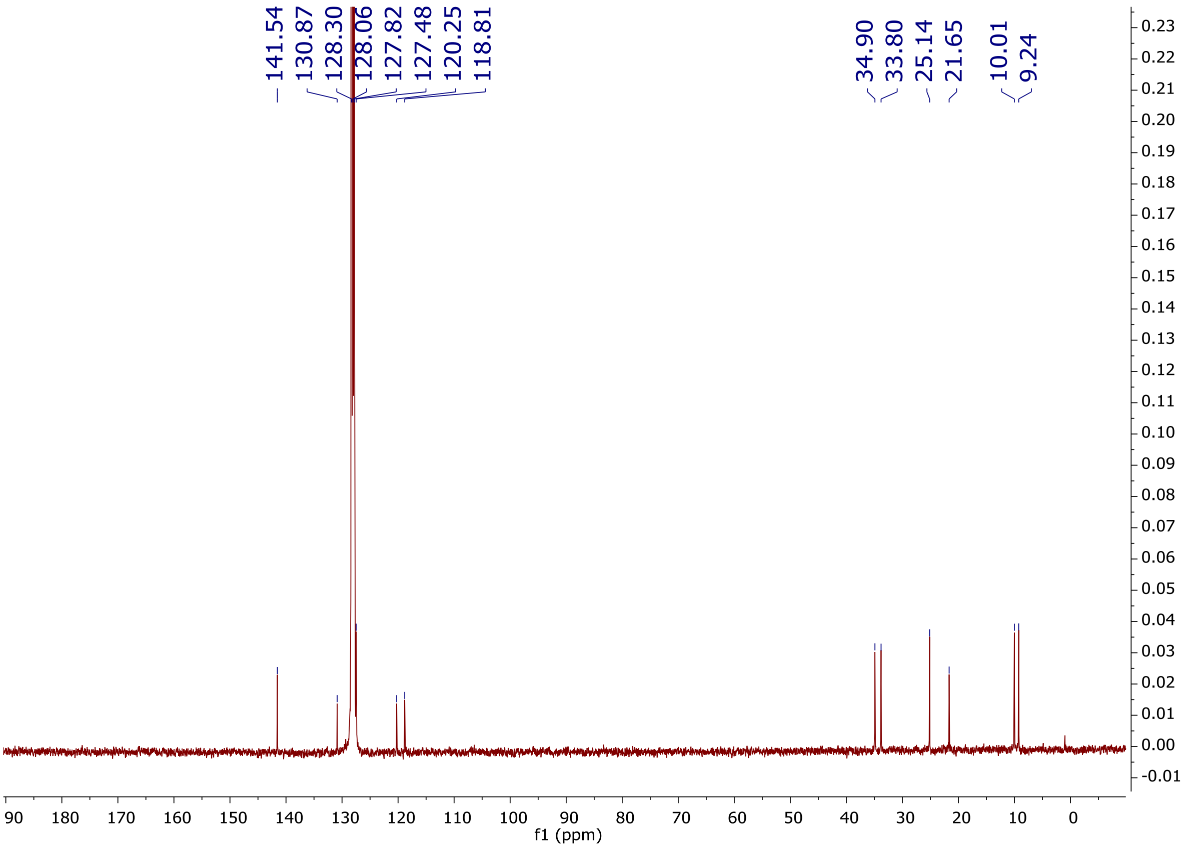

Supplementary Fig. 7. $^{13}C\{^{1}H\}$ NMR spectrum of **2** (in C_6D_6).

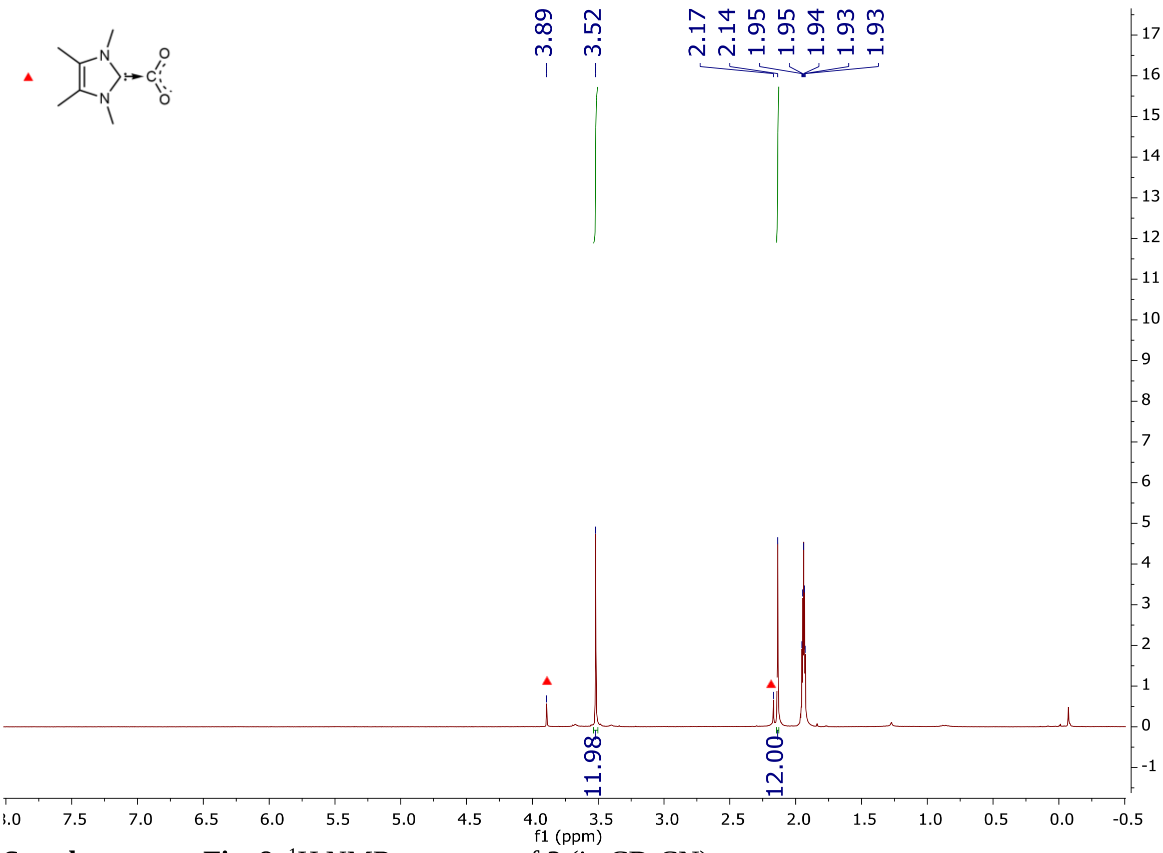

Supplementary Fig. 8. ^{1}H NMR spectrum of **3** (in CD_3CN).

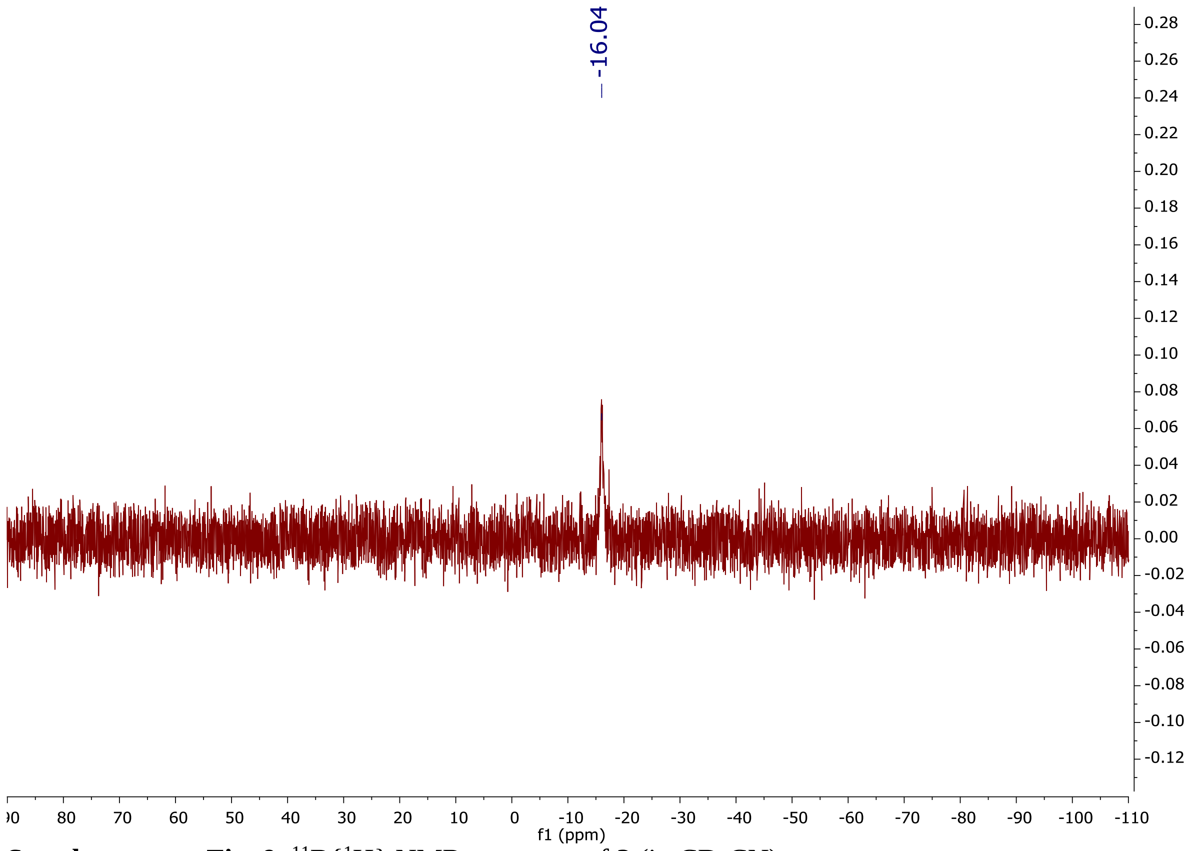

Supplementary Fig. 9. $^{11}B\{^{1}H\}$ NMR spectrum of **3** (in CD_3CN).

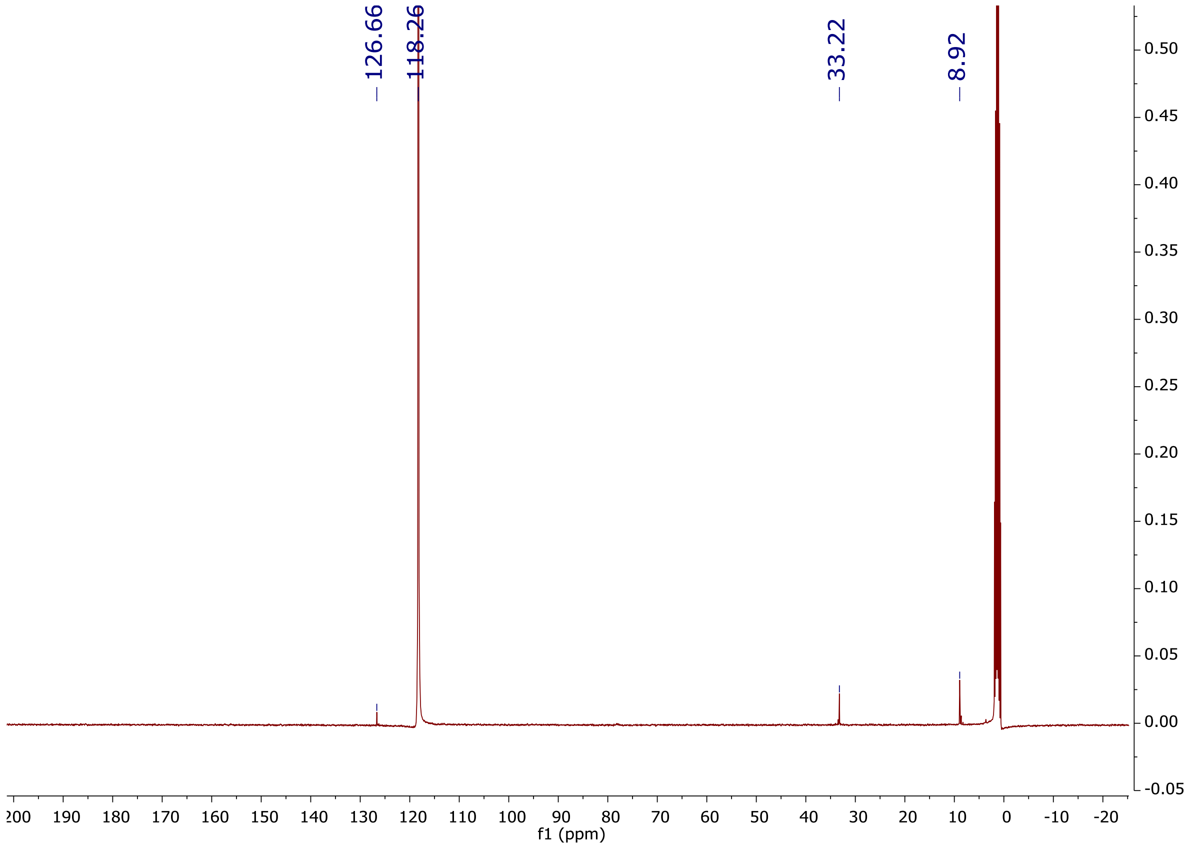

Supplementary Fig. 10. $^{13}C\{^{1}H\}$ NMR spectrum of **3** (in CD_3CN).

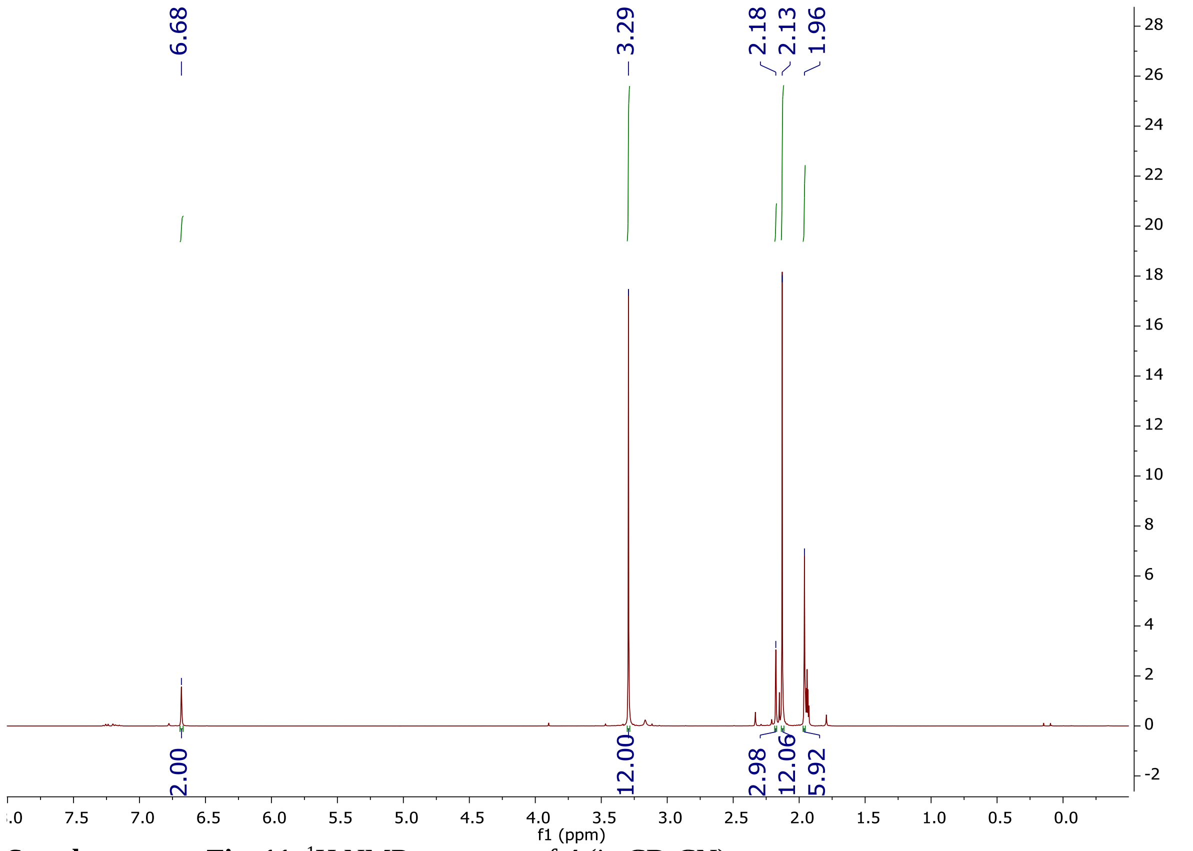

Supplementary Fig. 11. ^{1}H NMR spectrum of **4** (in CD_3CN).

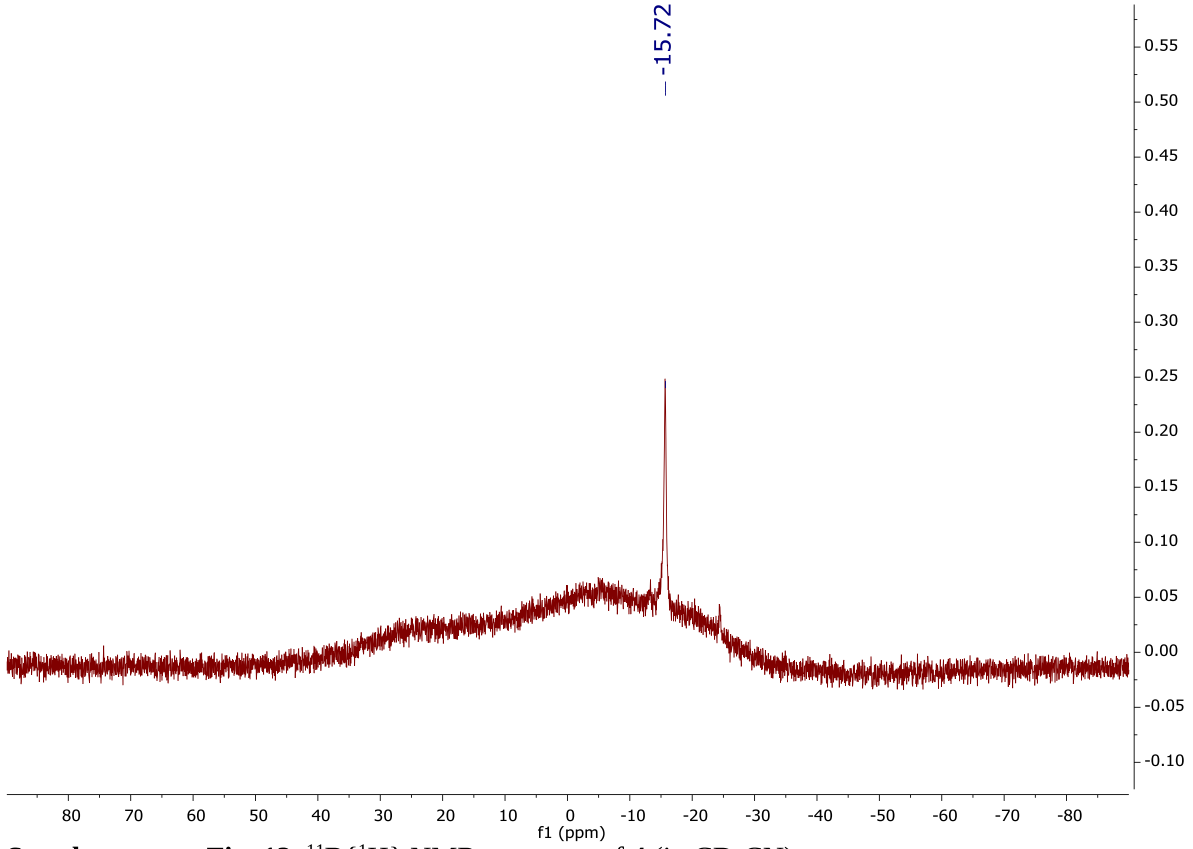

Supplementary Fig. 12. ^{11}B{^{1}H} NMR spectrum of **4** (in CD_3CN).

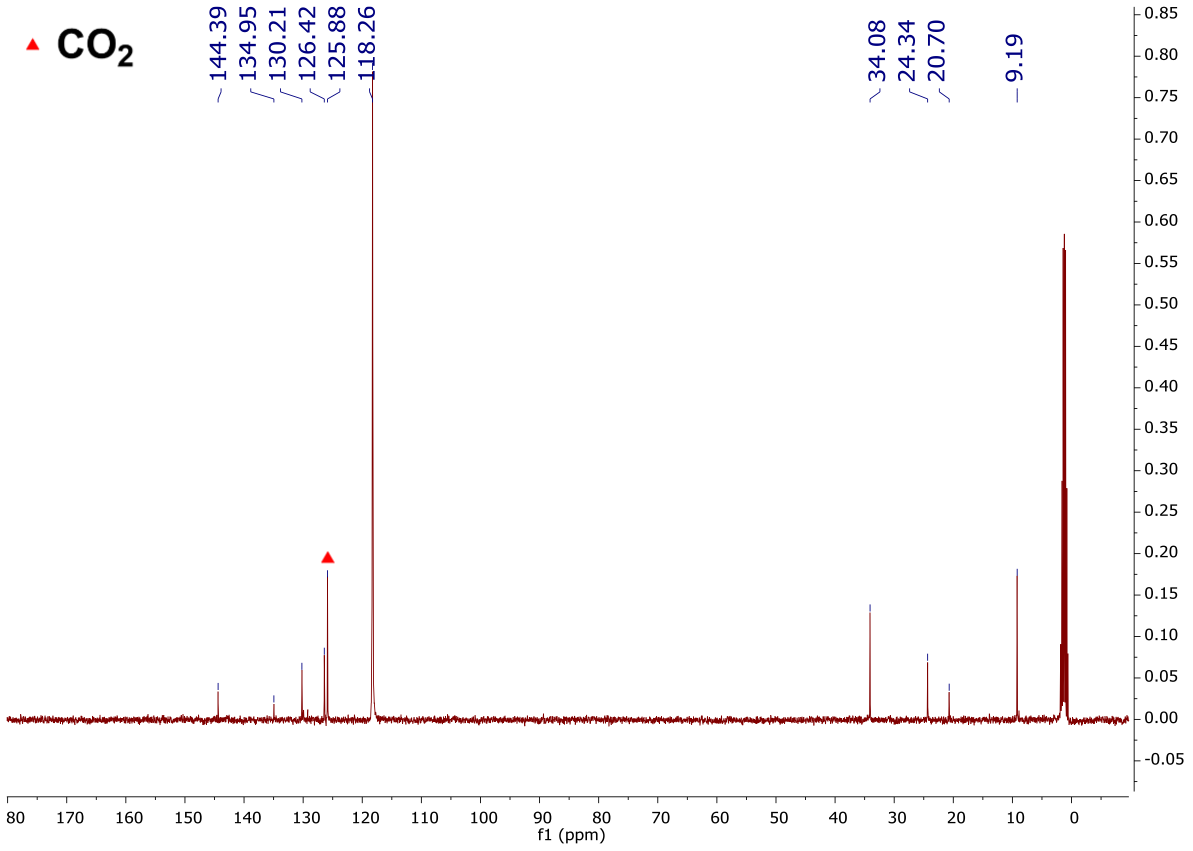

Supplementary Fig. 13. $^{13}C\{^{1}H\}$ NMR spectrum of **4** (in CD_3CN).

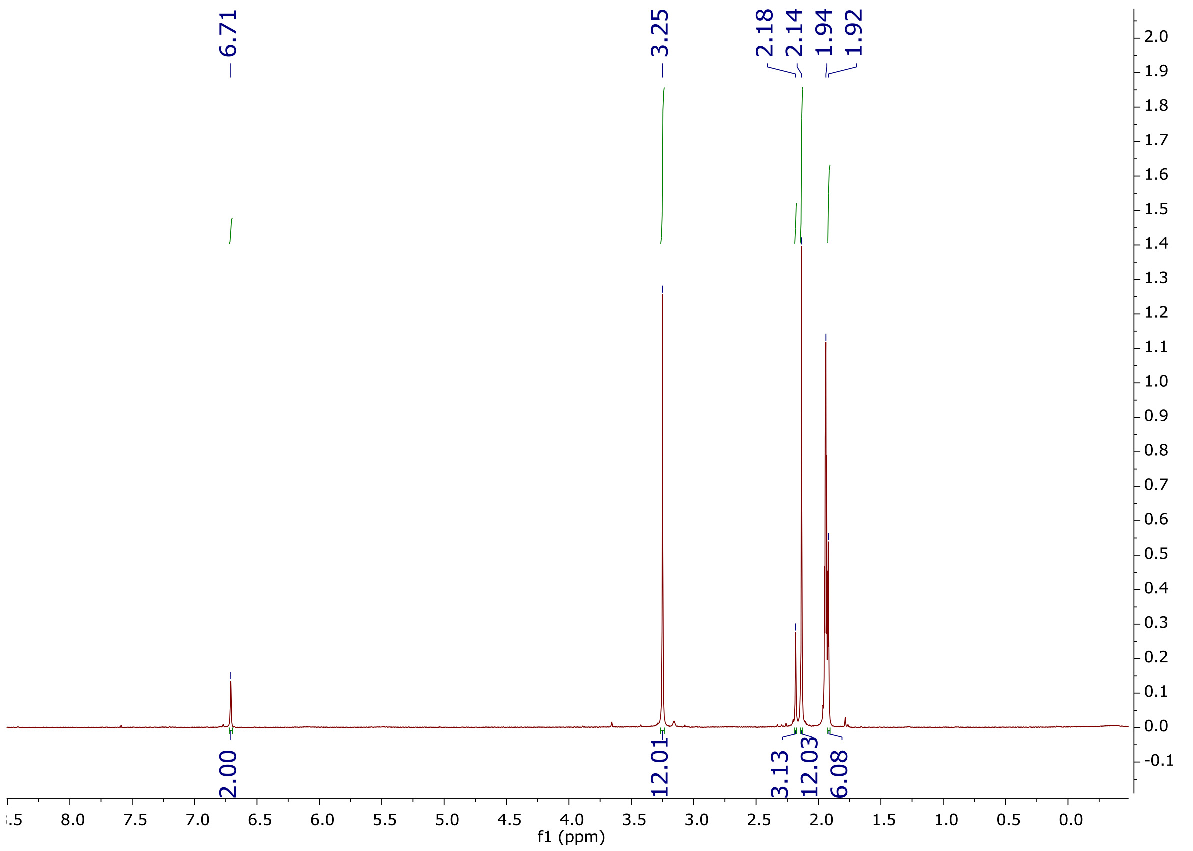

Supplementary Fig. 14. ^{1}H NMR spectrum of **4**·$B(OH)_3$ (in CD_3CN).

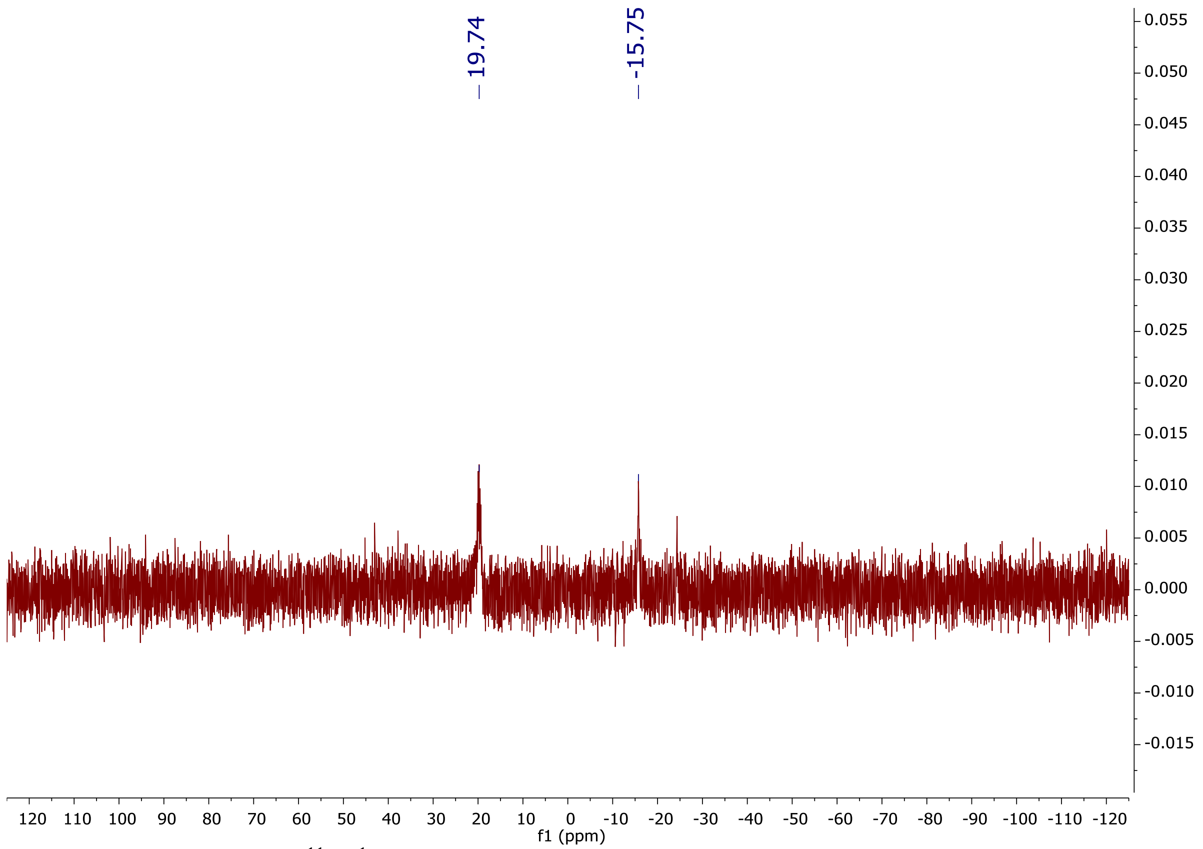

Supplementary Fig. 15. $^{11}B\{^{1}H\}$ NMR spectrum of **4**·B(OH)$_3$ (in CD_3CN).

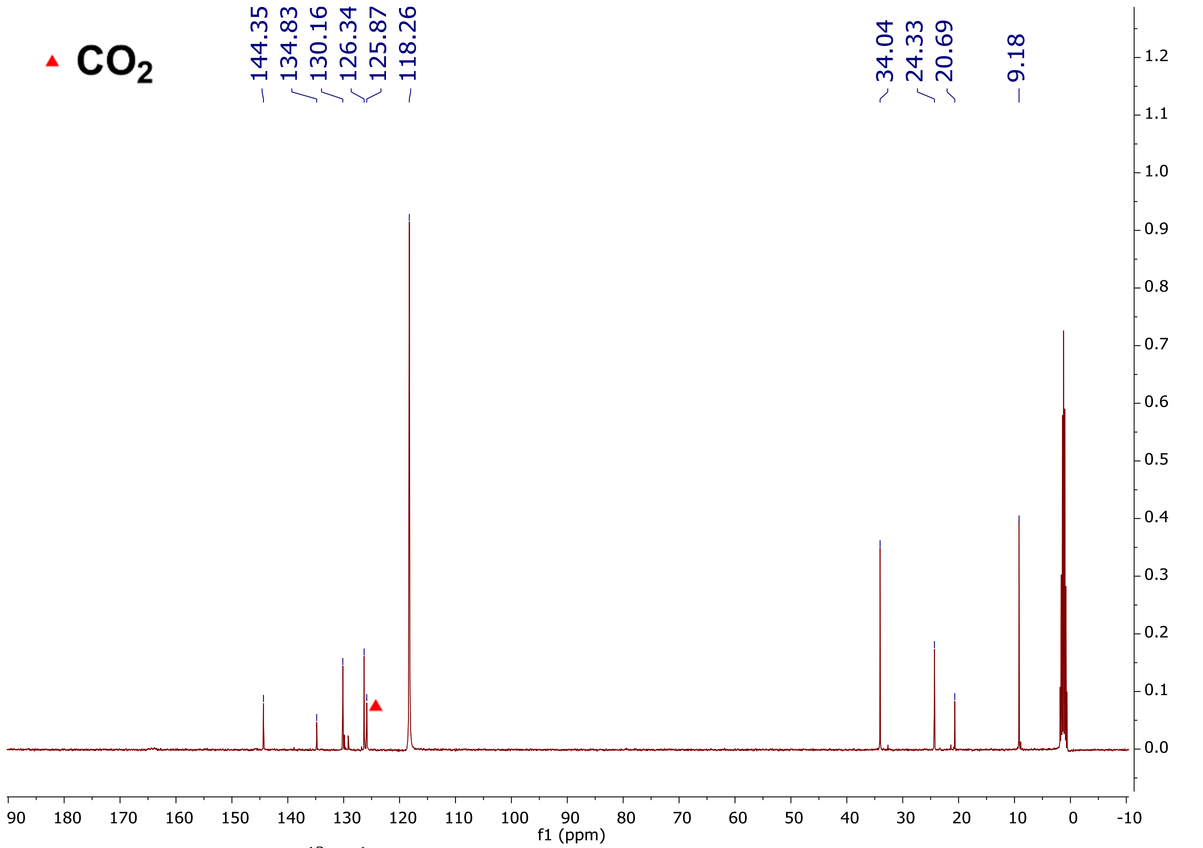

Supplementary Fig. 16. $^{13}C\{^{1}H\}$ NMR spectrum of **4**·B(OH)$_3$ (in CD_3CN).

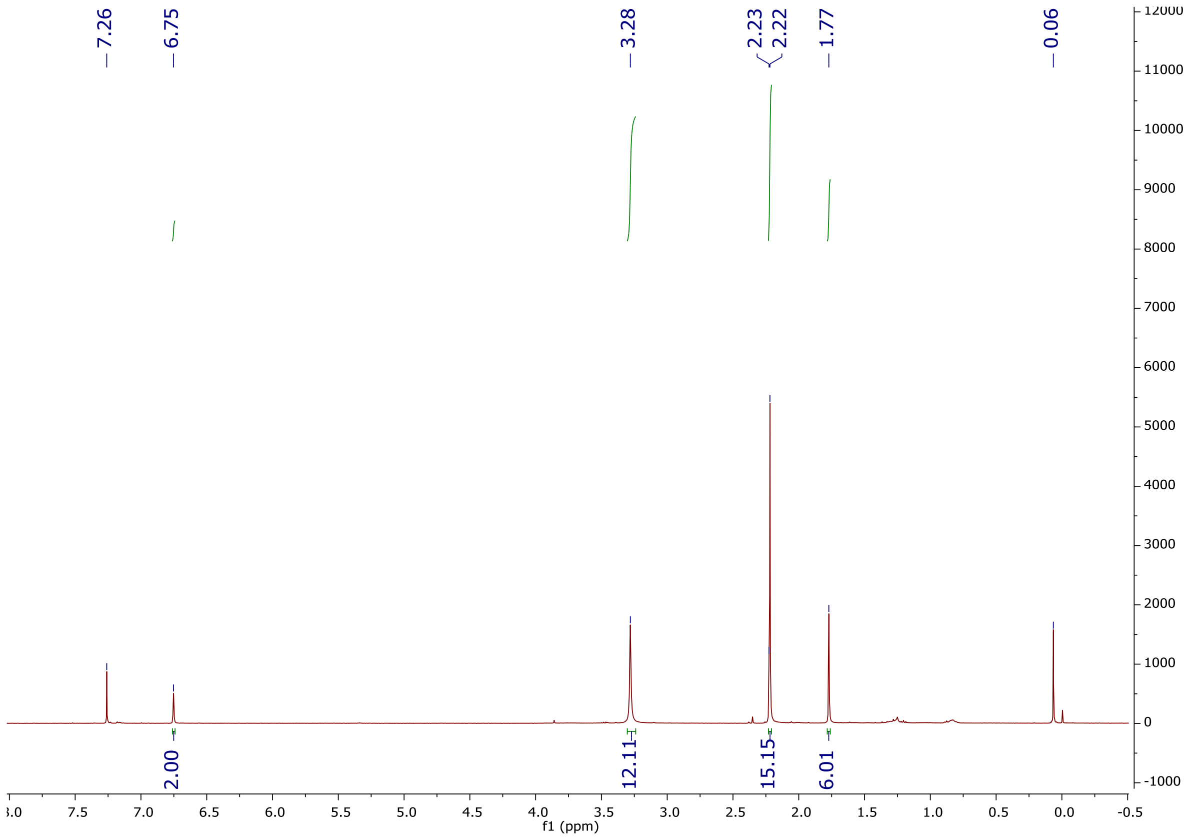

Supplementary Fig. 17. ^{1}H NMR spectrum of **5** (in $CDCl_3$).

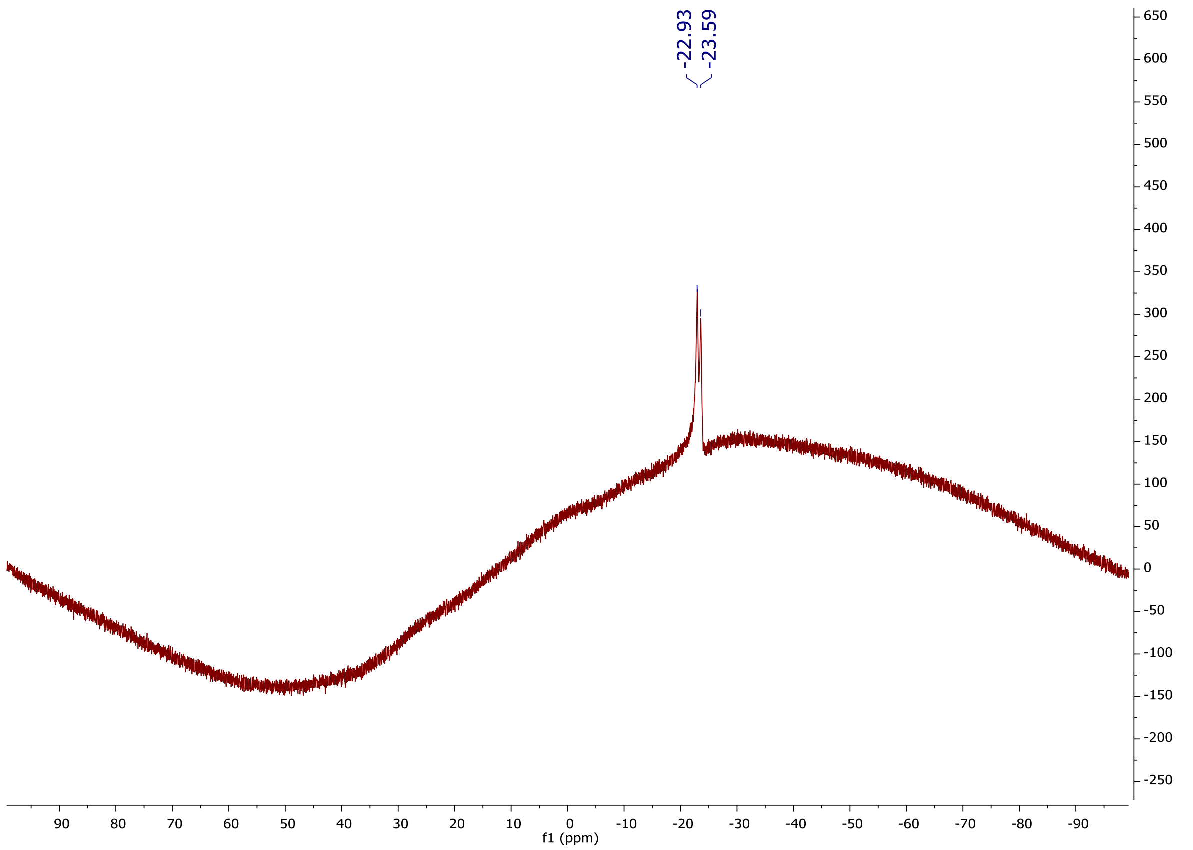

Supplementary Fig. 18. ^{11}B NMR spectrum of **5** (in $CDCl_3$).

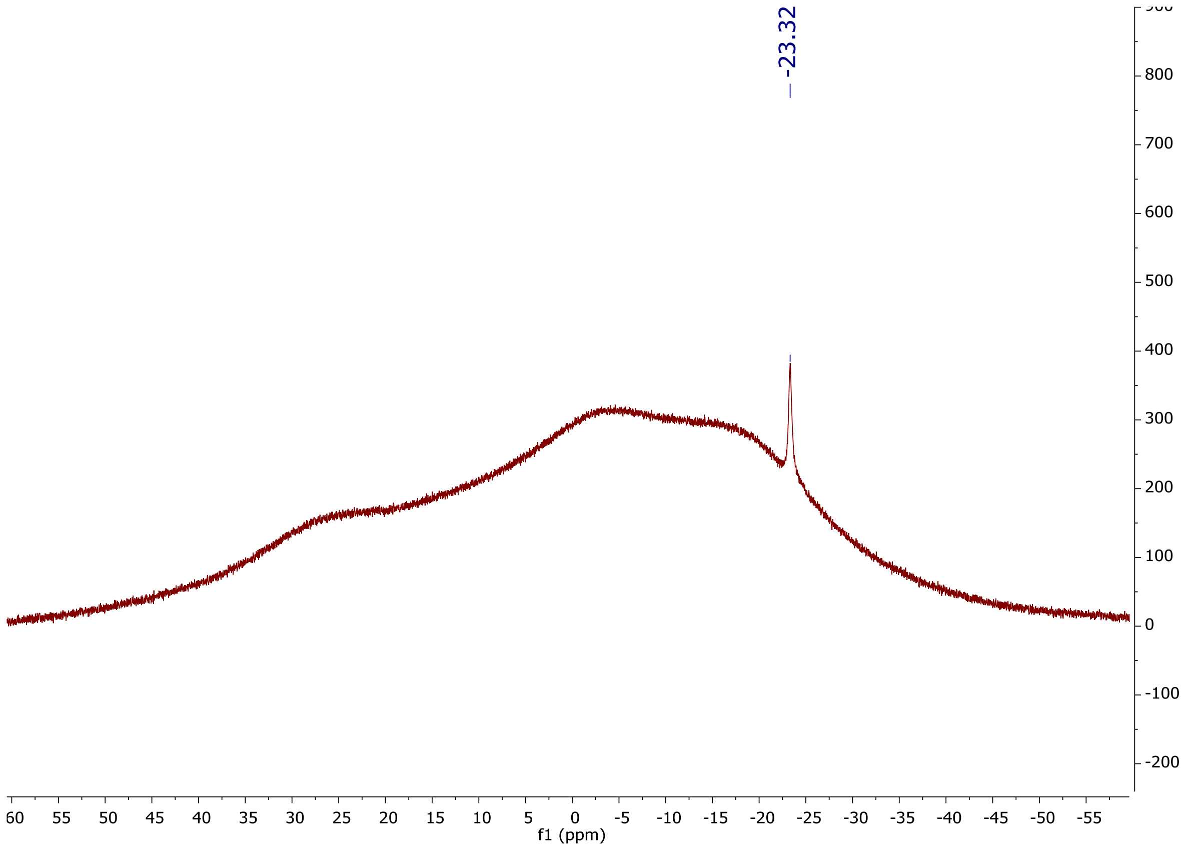

Supplementary Fig. 19. $^{11}B\{^{1}H\}$ NMR spectrum of **5** (in $CDCl_3$).

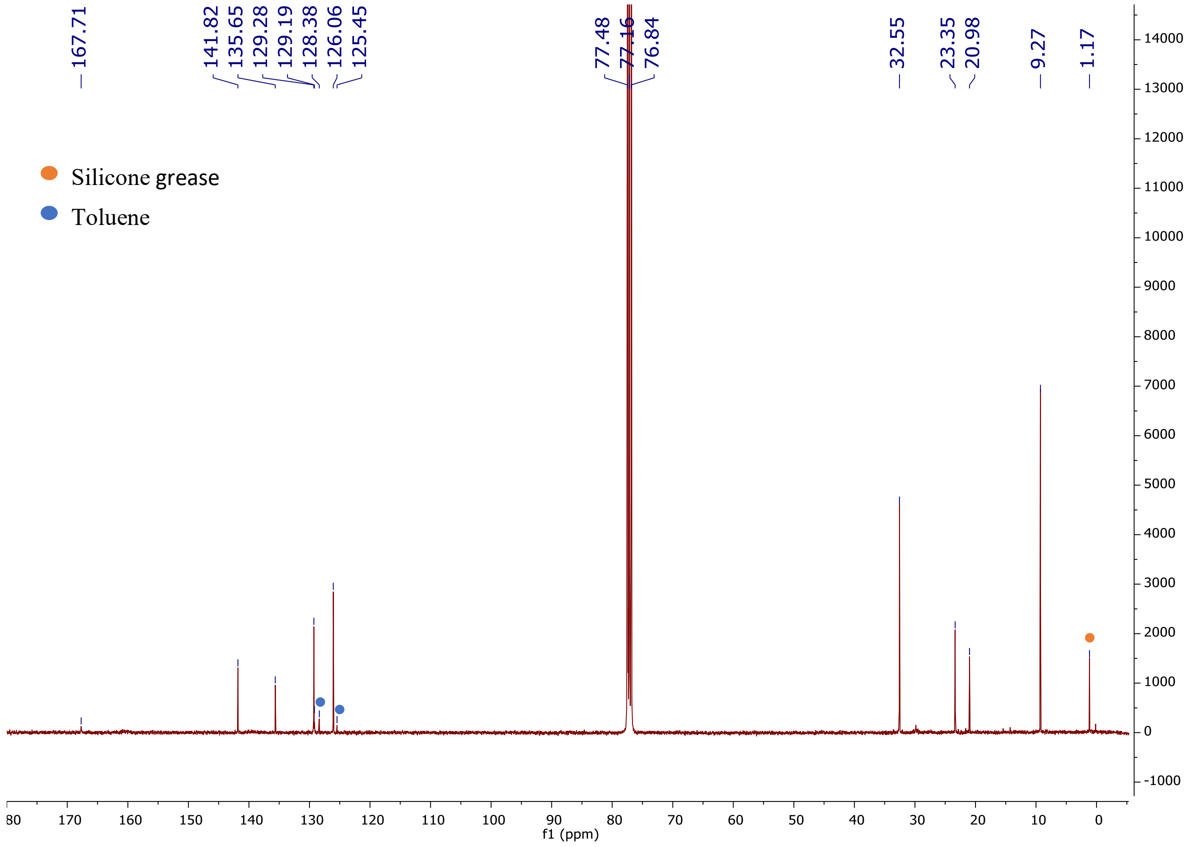

Supplementary Fig. 20. $^{13}C\{^{1}H\}$ NMR spectrum of **5** (in $CDCl_3$).

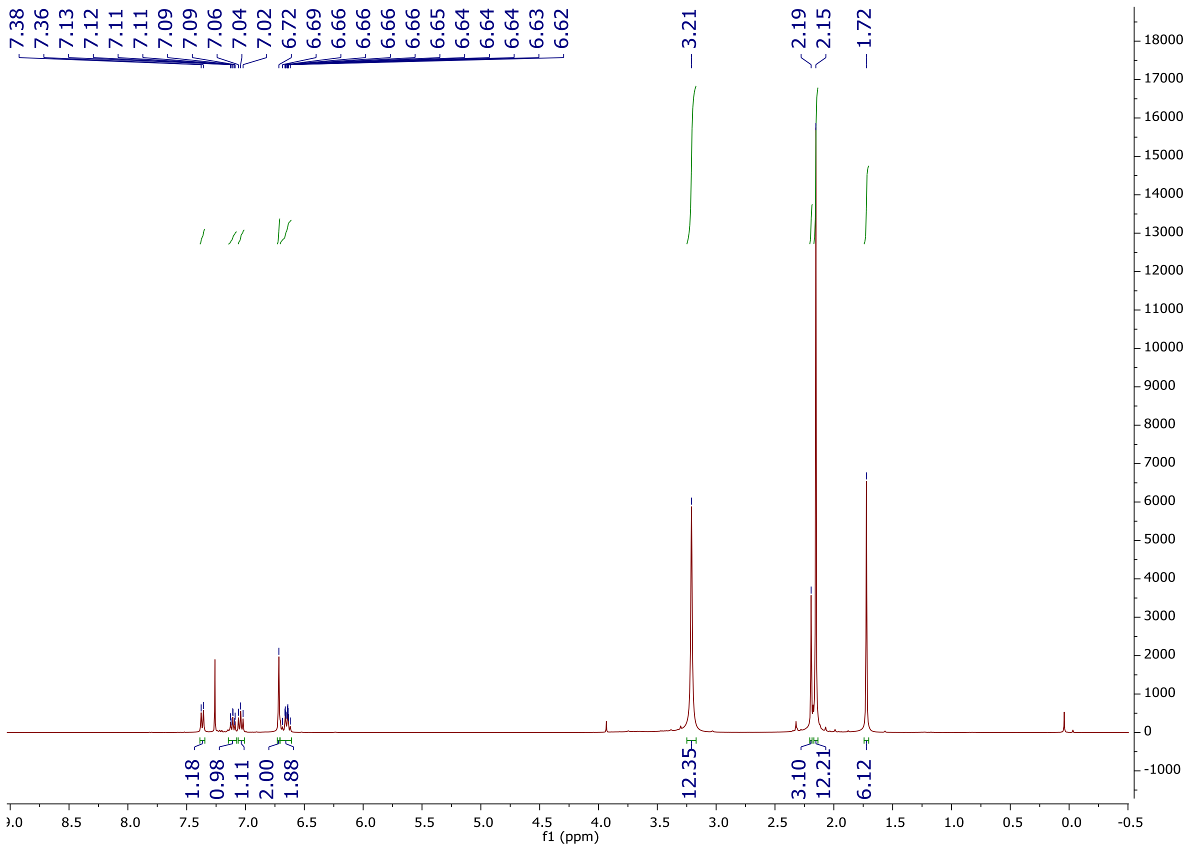

Supplementary Fig. 21. ^{1}H NMR spectrum of **6** (in $CDCl_3$).

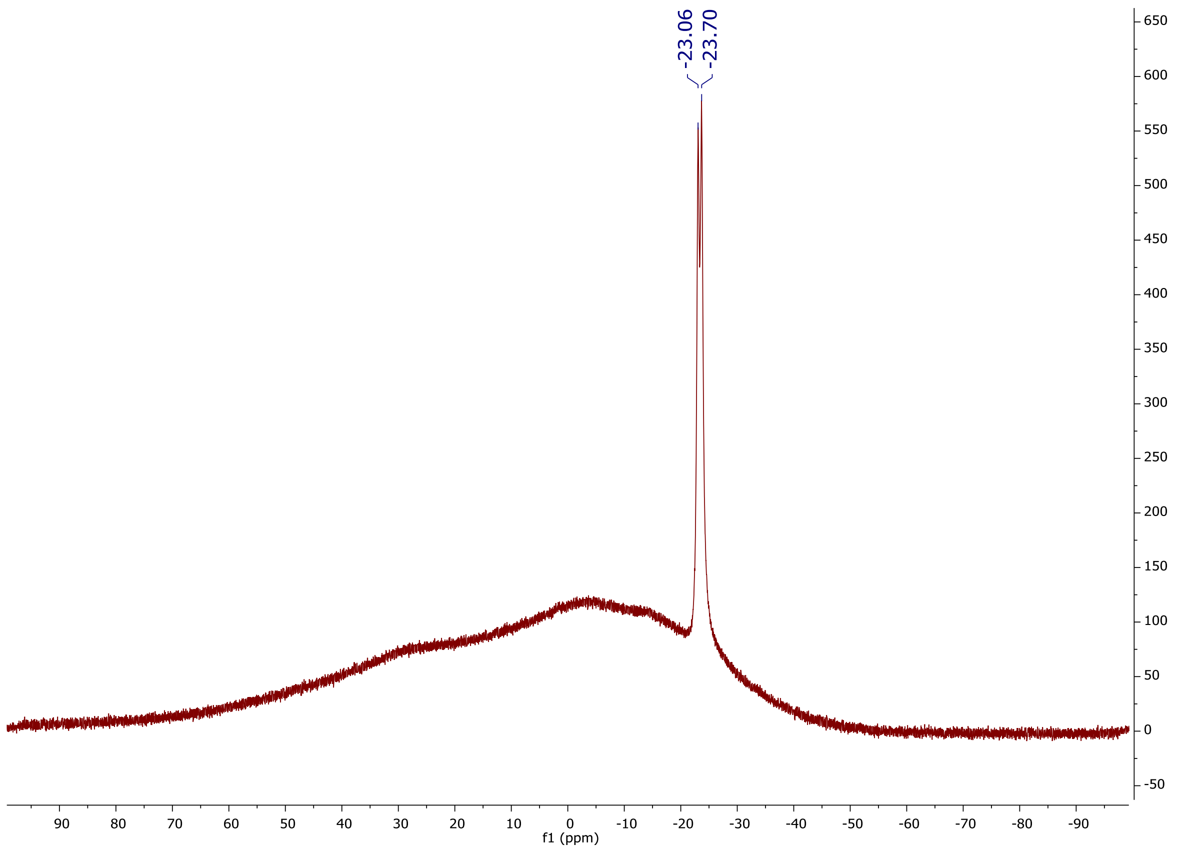

Supplementary Fig. 22. ^{11}B NMR spectrum of **6** (in $CDCl_3$).

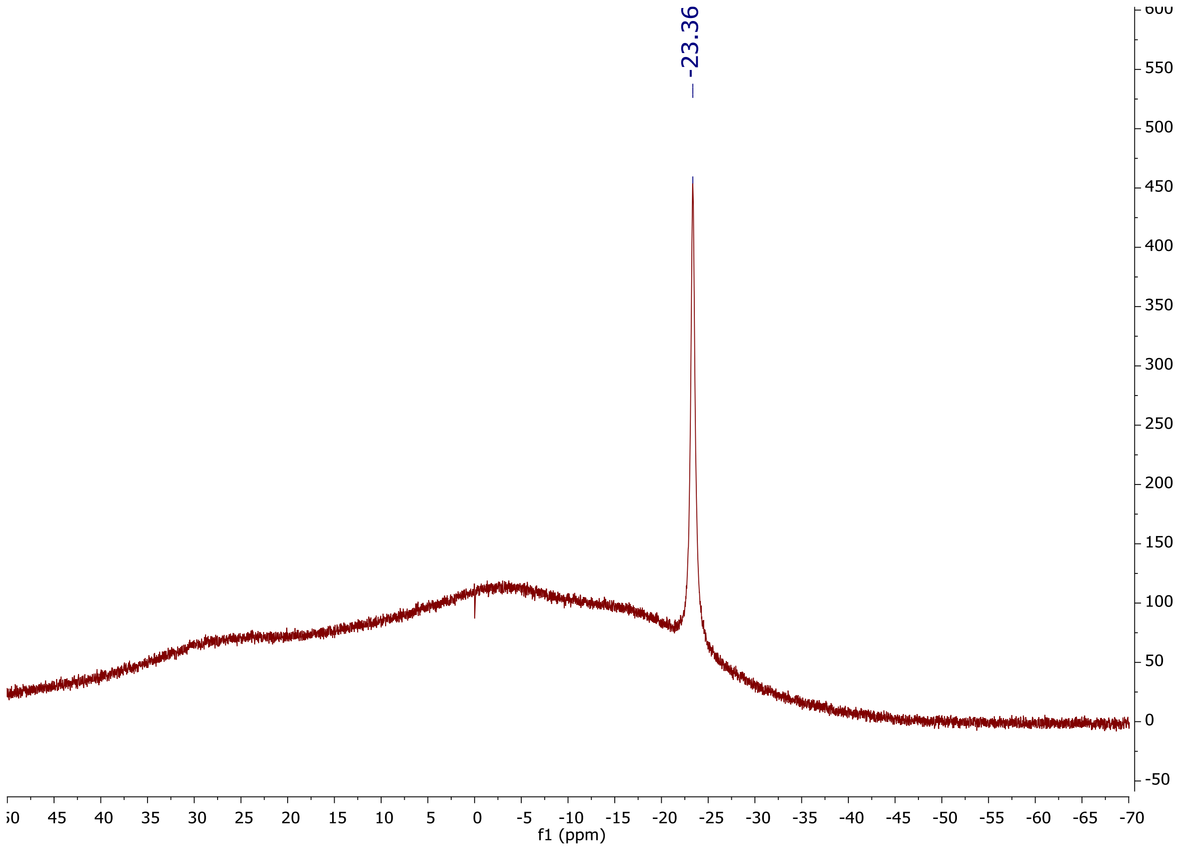

Supplementary Fig. 23. $^{11}B\{^{1}H\}$ NMR spectrum of **6** (in $CDCl_3$).

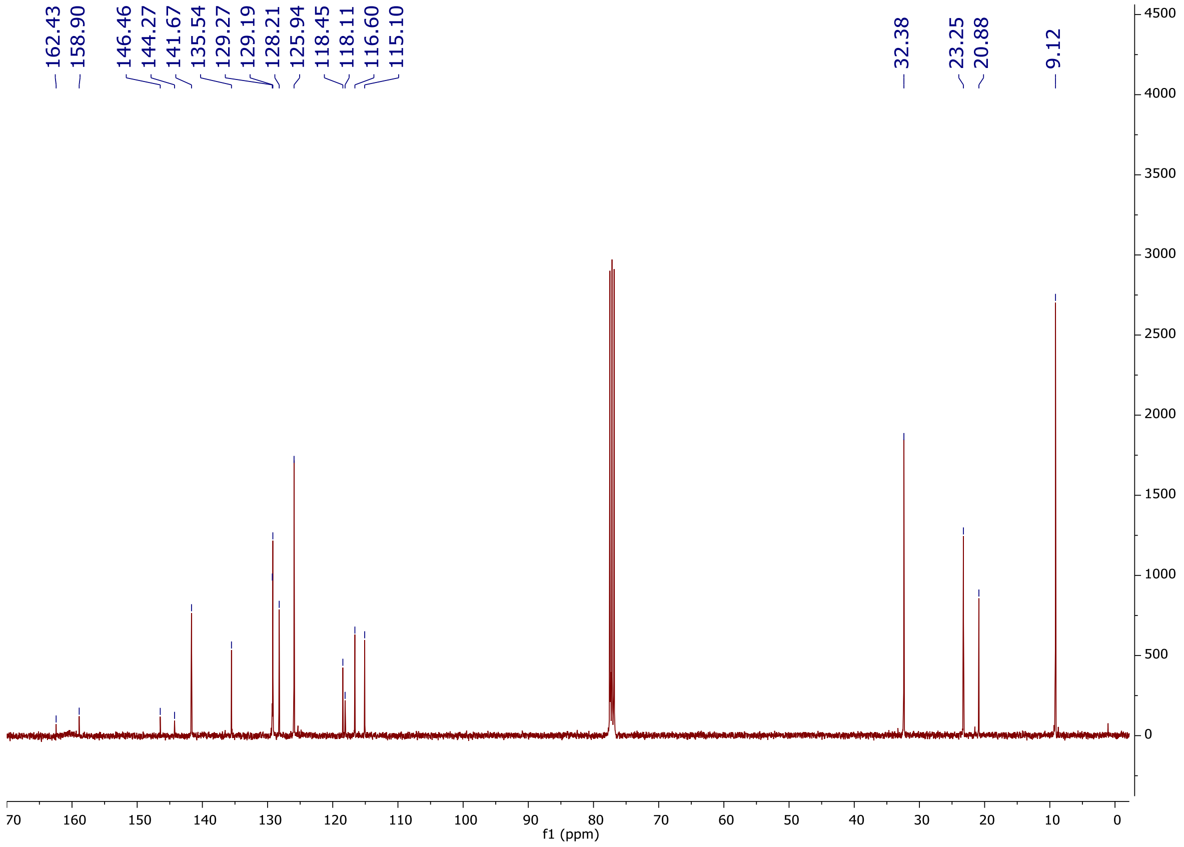

Supplementary Fig. 24. $^{13}C\{^{1}H\}$ NMR spectrum of **6** (in $CDCl_3$).

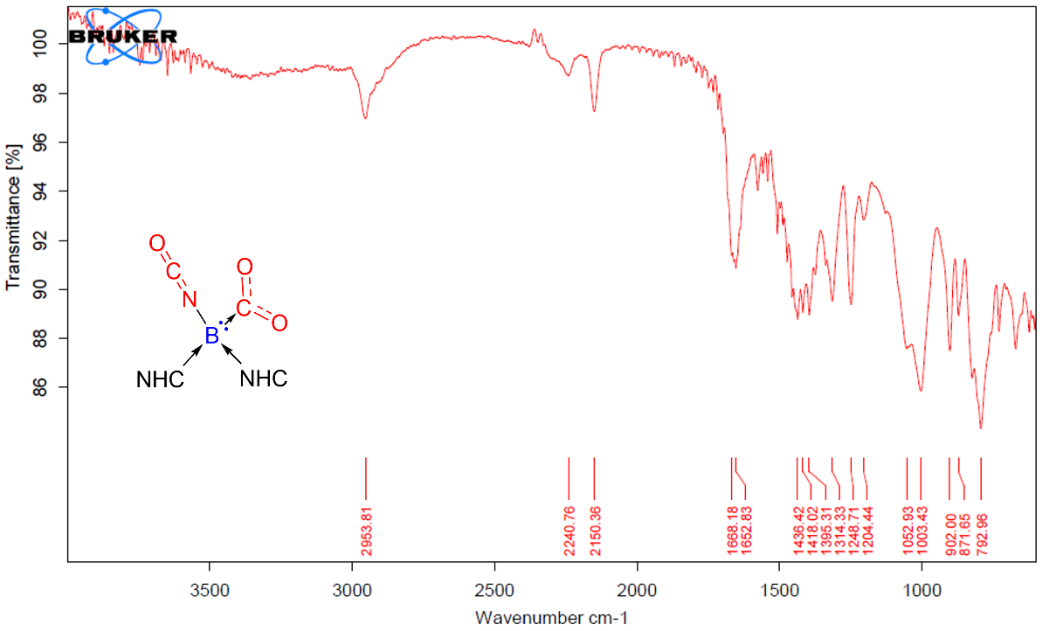

Supplementary Fig. 25. FT-IR spectra of **3**.

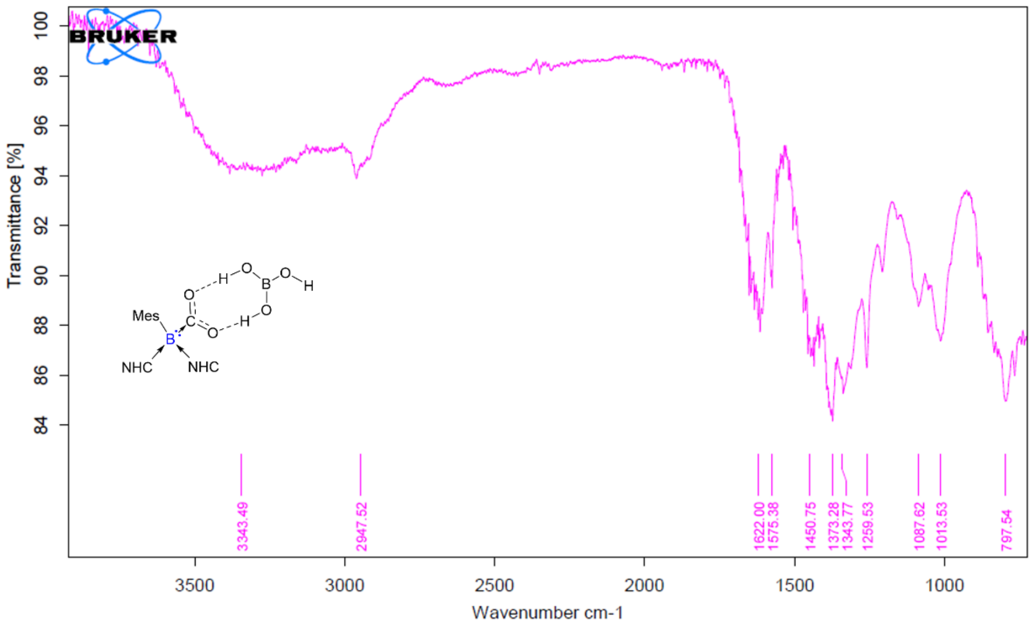

Supplementary Fig. 26. FT-IR spectra of **4**·B(OH)3.

2. Supplementary Discussion

X-Ray Crystallography Data: The X-ray diffraction intensity data were measured at 100 K with a Bruker APEX II diffractometer equipped with a CCD detector, employing Mo K α radiation (λ = 0.71073 Å), with the SMART suite of programs.[3] All data were processed and corrected for Lorentz and polarization effects with SAINT and for absorption effects with SADABS. Structural solution and refinement were carried out with the SHELXTL suite of programs.[4] The structures were solved by direct methods to locate the heavy atoms, followed by difference maps for the light, non-hydrogen atoms. Details of the crystallographic data and a summary of the intensity data collection parameters for **1-6** are listed in Supplementary Tables 1 and 2.

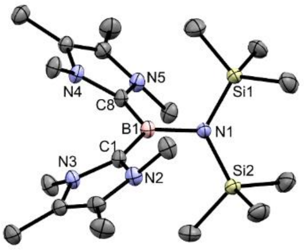

Supplementary Fig. 27. X-ray crystal structure of **1** with thermal ellipsoids shown at 50% probability. All hydrogen atoms are removed for clarity. Selected bond lengths (Å) and angles (deg): B1-C1 1.508(2), B1-C8 1.509(2), B1-N1 1.5327(19), C1-B1-C8 119.28(13), C1-B1-N1 120.63(13), C8-B1-N1 120.09(13). CCDC deposition number 2235472.

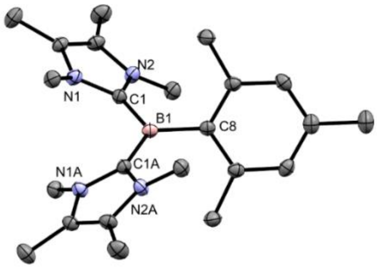

Supplementary Fig. 28. X-ray crystal structure of **2** with thermal ellipsoids shown at 50% probability. All hydrogen atoms are removed for clarity. Selected bond lengths (Å) and angles (deg): B1-C1 1.520(3), B1-C1A 1.519(3), B1-C8 1.588(5), C1A-B1-C8 120.99(15), C1-B1-C8 120.99(15), C1-B1-C1A 118.0(3). CCDC deposition number 2257718.

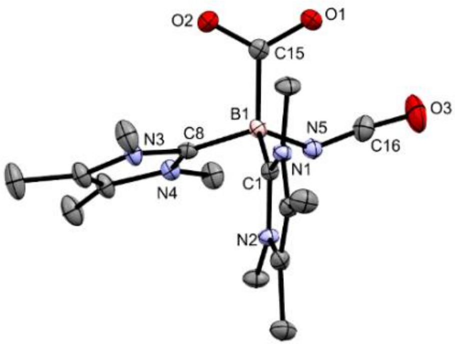

Supplementary Fig. 29. X-ray crystal structure of **3** with thermal ellipsoids shown at 50% probability. All hydrogen atoms are removed for clarity. Selected bond lengths (Å) and angles (deg): B1-C15 1.645(3), C1-B1 1.640(3), C8-B1 1.639(3), B1-N5 1.541(3), C8-B1-C1 106.55(17), C1-B1-N5 105.65(18), N5-B1-C8 111.13(17), O1-C15-O2 123.8(2). CCDC deposition number 2235466.

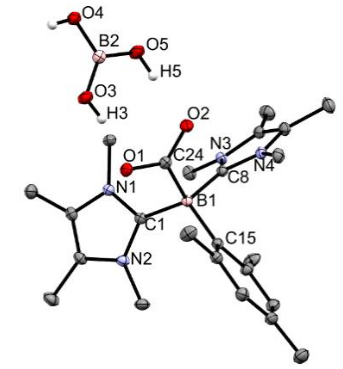

Supplementary Fig. 30. X-ray crystal structure of **4**·B(OH)$_3$ with thermal ellipsoids shown at 50% probability. All hydrogen atoms except for those on boric acid are removed for clarity. Selected bond lengths (Å) and angles (deg): C1-B1 1.6532(16), C8-B1 1.6514(17), C15-B1 1.6608(17), C24-B1 1.6810(17), C24-O1 1.2790(14), C24-O2 1.2643(14), C1-B1-C8 114.24(9), C1-B1-C15 112.85(9), C8-B1-C15 106.30(9), O1-C24-O2 121.60(11), O1-C24-B1 119.45(10), O2-C24-B1 118.68(10). CCDC deposition number 2257719.

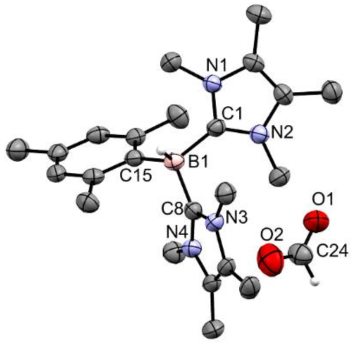

Supplementary Fig. 31. X-ray crystal structure of **5** with thermal ellipsoids shown at 50% probability. All hydrogen atoms except for those on the boron and formate are removed for clarity. Selected bond lengths (Å) and angles (deg): C1-B1 1.635(4), C8-B1 1.621(4), C15-B1 1.622(4), C24-O1 1.223(4), C24-O2 1.210(4), O1-C24-O2 133.6(4), C1-B1-C15 117.2(2), C1-B1-C8 108.4(2), C8-B1-C15 116.3(2). CCDC deposition number 2307337.

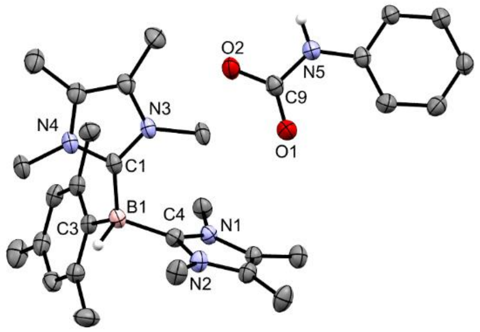

Supplementary Fig. 32. X-ray crystal structure of **6** with thermal ellipsoids shown at 50% probability. All hydrogen atoms except for those on the boron or carbamate are removed for clarity. Selected bond lengths (Å) and angles (deg): C1-B1 1.618(3), C4-B1 1.631(3), C3-B1 1.643(3), C9-O1 1.246(2), C9-O2 1.268(2), C9-N5 1.406(2), C1-B1-C4 111.65(15), C1-B1-C3 115.09(16), C3-B1-C4 111.65(15), O1-C9-O2 126.14(18), O1-C9-N5 119.53(17), O2-C9-N5 114.33(17). CCDC deposition number 2307338.

Supplementary Table 1. X-Ray data for compounds **1, 2** and **3.**

	**1**	**2**	**3**
Formula	$C_{20}H_{42}BN_5Si_2$	$C_{23}H_{35}BN_4$	$C_{16}H_{24}BN_5O_3$
Fw	419.57	378.36	345.21
T/K	100(2)	100(2)	100(2)
cryst system	monoclinic	orthorhombic	monoclinic
space group	*P* 1 2/*n* 1	*A b a* 2	*P* 1 21/*c* 1
a (Å)	16.4977(9)	16.9865(10)	12.5921(6)
b (Å)	10.3490(6)	9.6158(6)	16.8292(6)
c (Å)	16.9174(11)	13.2699(7)	8.4177(4)
α (deg)	90	90	90
β (deg)	118.115(2)	90	94.529(2)
γ (deg)	90	90	90
V (Å^3)	2547.6(3)	2167.5(2)	1778.27(14)
Z	4	4	4
d_{calcd} (g cm^{-3})	1.094	1.159	1.289
μ (mm^{-1})	0.154	0.069	0.090
F(000)	920	824	736
cryst size (mm)	0.20 x 0.24 x 0.26	0.12 x 0.18 x 0.24	0.01 x 0.02 x 0.12
2θ range (deg)	4.83 < 2θ < 60.09	4.796 < 2θ < 62.05	5.424 < 2θ <52.74
index range	$-23 \le h \le 26$, $-16 \le k \le 16$, $-26 \le l \le 26$	$-24 \le h \le 24$, $-13 \le k \le 13$, $-19 \le \mathrm{l} \le 19$	$-15 \le h \le 15$, $-21 \le k \le 18$, $-10 \le l \le 10$
no. of rflns collected	67803	25498	20833
no. of indep rflns	10664	3461	3649
*R*1, *wR*2 ($I > 2\sigma(I)$)	0.0606, 0.1229	0.0490, 0.0984	0.0584, 0.1175
*R*1, *wR*2 (all data)	0.1340, 0.1491	0.0823, 0.1135	0.0854, 0.1294
goodness of fit, F^2	1.036	1.025	1.048
no. of data/restraints/params	10664 / 0 / 267	3461 / 1 / 134	3649 / 0 / 234
largest diff peak and hole, eÅ^{-3}	0.429 / -0.429	0.236 / -0.220	0.411 and -0.240

Supplementary Table 2. X-Ray data for compound **4**, **5**, and **6.**

	**4·B(OH)$_3$.(THF)$_1$**	**5**	**6**
Formula	$C_{28}H_{46}B_2N_4O_6$	$C_{24}H_{37}BN_4O_2$	$C_{30}H_{42}BN_5O_2$
Fw	556.31	424.38	515.49
T/K	100(2)	100(2)	100(2)
cryst system	triclinic	monoclinic	monoclinic
space group	P -1	P 1 21/c 1	P 1 21/n 1
a (Å)	8.8685(4)	11.0959(6)	16.0712(7)
b (Å)	10.2532(4)	10.8210(6)	8.3268(4)
c (Å)	17.2158(6)	19.8676(11)	21.7280(9)
α (deg)	91.7807(15)	90	90
β (deg)	93.5355(16)	96.672(3)	100.767(3)
γ (deg)	111.7585(14)	90	90
V (Å^3)	1448.69(10)	2369.3(2)	2856.5(2)
Z	2	4	4
d_{calcd} (g cm^{-3})	1.275	1.190	1.199
μ (mm^{-1})	0.088	0.595	0.594
F(000)	600	920	1112
cryst size (mm)	0.08 x 0.20 x 0.22	0.14 x 0.22 x 0.42	0.06 x 0.12 x 0.22
2θ range (deg)	4.776 < 2θ < 68.39	8.022 < 2θ < 134.2	6.310 < 2θ < 134.6
index range	$-14 \le h \le 14$<br>$-16 \le k \le 16$,<br>$-27 \le l \le 27$	$-10 \le h \le 13$,<br>$-12 \le k \le 13$,<br>$-23 \le l \le 23$	$-19 \le h \le 19$,<br>$-9 \le k \le 9$,<br>$-25 \le l \le 24$
no. of rflns collected	51241	16065	23016
no. of indep rflns	12109	4213	5019
R1, wR2 ($I > 2\sigma(I)$)	0.0575, 0.1303	0.0812, 0.2071	0.0485, 0.1251
R1, wR2 (all data)	0.1021, 0.1495	0.1019, 0.2343	0.0630, 0.1359
goodness of fit, F^2	1.037	1.036	1.069
no. of data/restraints/params	12109 / 0 / 375	4213 / 0 / 295	5019 / 0 / 358
largest diff peak and hole, eÅ^{-3}	0.465 / -0.321	0.404 / -0.378	0.214 / -0.279

Computational Studies

All geometry optimizations, MO and NBO analysis were performed at the M06-2X[5]/def2-TZVP[6] level of theory using the Gaussian 16 C.01 program.[7] The Polarizable Continuum Model (PCM) using the integral equation formalism variant (IEFPCM)[8-10] is applied as a solvation model. In order to match the actual experimental situations, we used toluene as the solvent in the present theoretical calculations.The mechanism for the formation of compound **3** by the reaction of compound **1** with CO_2 was computed using the M06-2X/def2-TZVP/ IEFPCM(toluene) level of theory. The mechanism for the formation of compound **5** by the reaction of compound **4** with NH_3BH_3 was computed using the M06-2X/def2-TZVP/ IEFPCM(toluene) level of theory. The vibrational frequency calculations were performed to establish the nature of stationary points. The local minima were confirmed by zero imaginary frequencies and transition states were characterized by an imaginary frequency. In the free energy profile, the Gibbs free energy (ΔG) was calculated at 298.15 K and 1 atm.

Supplementary Table 3. Natural bond orbital (NBO) analysis of **1** at M06-2X/def2-TZVP level.

Bond Type	Occupancy	Polarization	Hybridization	Bond Length (Å)	WBI
BC_1 σ	1.96	67.71% C_1<br>32.29% B	C_1: $sp^{1.17}$<br>B: $sp^{2.02}$	1.511	1.185
BC_2 σ	1.96	69.12% C_2<br>30.88% B	C_2: $sp^{1.17}$<br>B: $sp^{2.02}$	1.511	1.183
BN_1 σ	1.96	75.69% N_1<br>34.31% B	N_1: $sp^{1.76}$<br>B: $sp^{1.97}$	1.524	0.789
Lone pair-B	0.93	---	$p^{1.00}$ (99.94%)	---	---

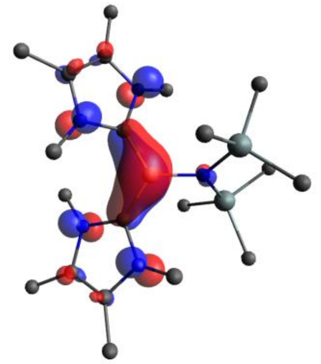

HOMO (-3.801 eV, Top View)

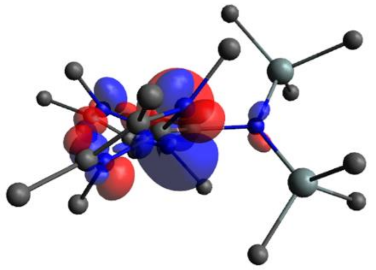

HOMO (Side View)

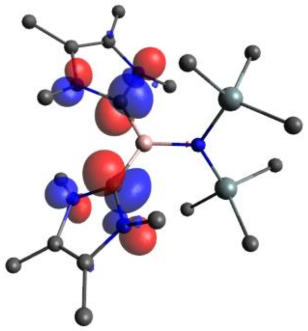

LUMO (0.885 eV, Top View)

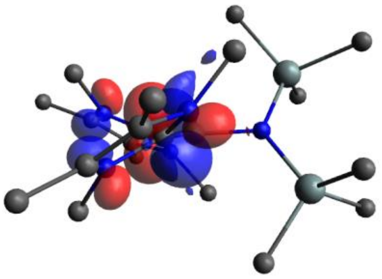

LUMO (Side View)

Supplementary Figure 33. Molecular orbitals of compound **1** (isovalue 0.03).

Supplementary Table 4. Natural bond orbital (NBO) analysis of **2** at M06-2X/def2-TZVP level.

Bond Type	Occupancy	Polarization	Hybridization	Bond Length (Å)	WBI
BC_1 σ	1.95	66.40% C_1<br>33.60% B	C_1: $sp^{1.16}$<br>B: $sp^{2.09}$	1.517	1.174
BC_2 σ	1.95	66.40% C_2<br>33.60% B	C_2: $sp^{1.16}$<br>B: $sp^{2.08}$	1.516	1.176
BC_2 π	1.55	51.14% C_2<br>48.86% B	C_2: $p^{1.00}$<br>B: $p^{1.00}$		
BC_3 σ	1.94	64.77% C_3<br>35.23% B	C_3: $sp^{1.79}$<br>B: $sp^{1.84}$	1.586	0.949

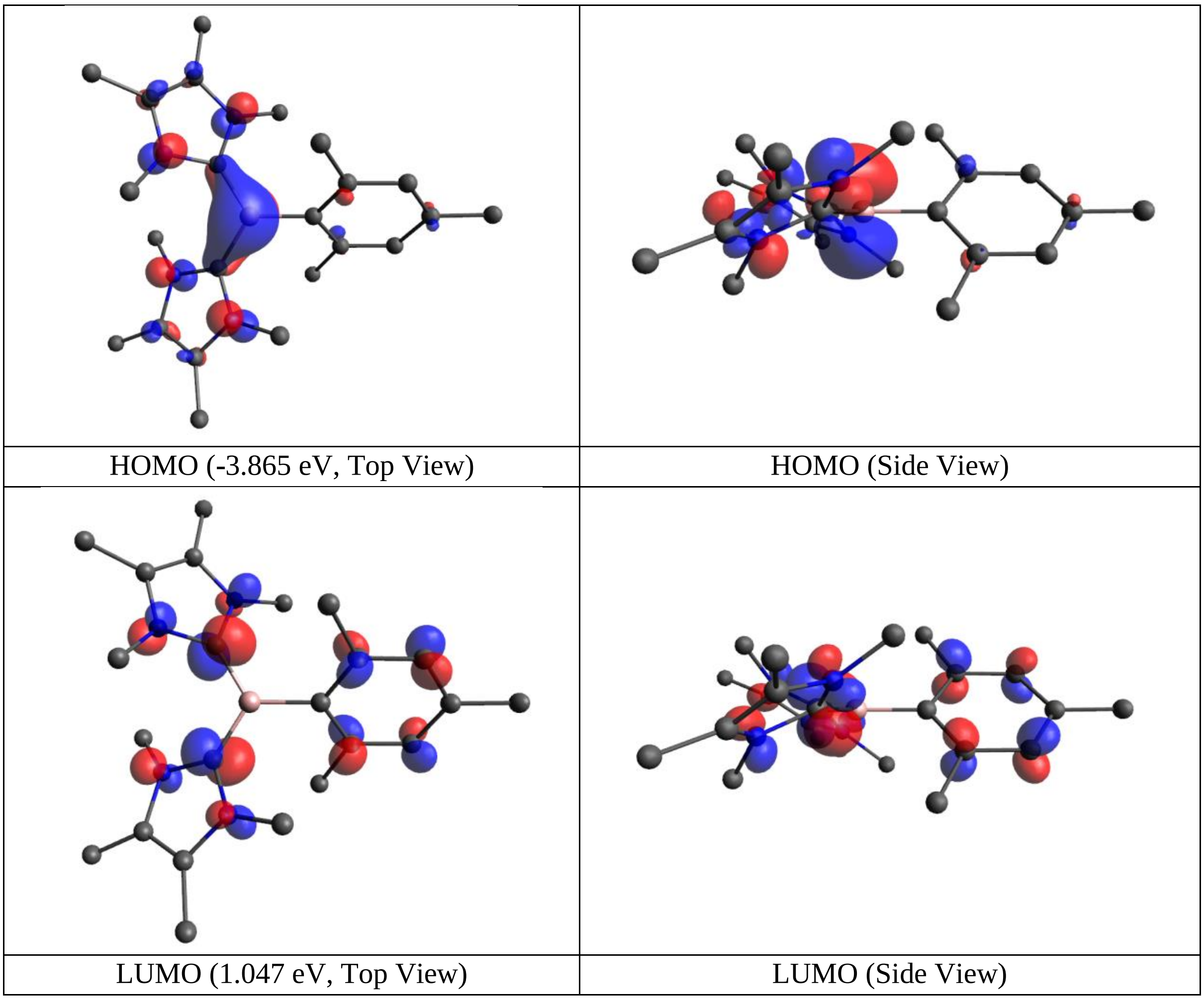

Supplementary Figure 34. Molecular Orbitals of **2** (isovalue 0.03).

Supplementary Table 5. Natural bond orbital (NBO) analysis of **3** at M06-2X/def2-TZVP level.

Bond Type	Occupancy	Polarization	Hybridization	Bond Length (Å)	WBI
BC_1 σ	1.95	66.53% C_1<br>33.47% B	C_1: $sp^{1.37}$<br>B: $sp^{3.04}$	1.629	0.853
BC_2 σ	1.95	66.37% C_2<br>33.63% B	C_2: $sp^{1.37}$<br>B: $sp^{3.22}$	1.625	0.867
BN_1 σ	1.97	73.54% N_1<br>26.46% B	N_1: $sp^{0.79}$<br>B: $sp^{3.07}$	1.527	0.806
BC_3 σ	1.93	56.28% C_3<br>43.72% B	C_3: $sp^{1.82}$<br>B: $sp^{2.70}$	1.647	0.860

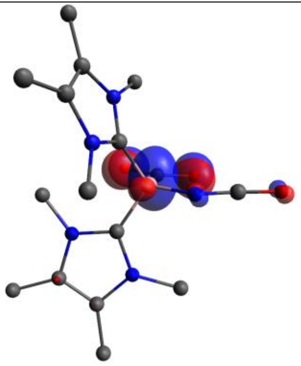	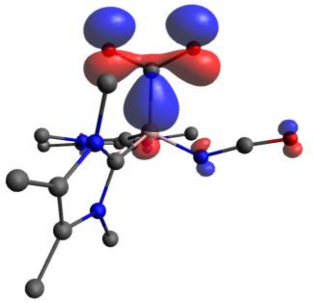
HOMO (-6.329 eV, Top View)	HOMO (Side View)
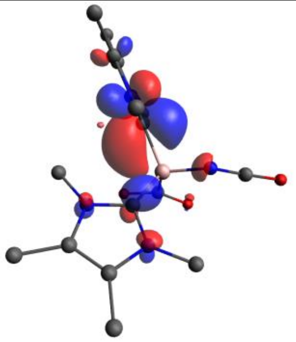	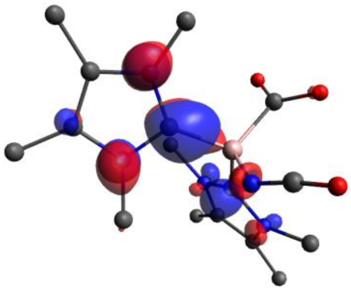
LUMO (0.637 eV, Top View)	LUMO (Side View)

Supplementary Figure 35. Molecular orbitals of compound **3** (isovalue 0.03).

**Compound**	Grouped NPA data
**1**	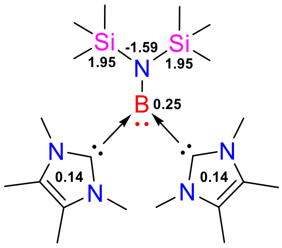
**3**	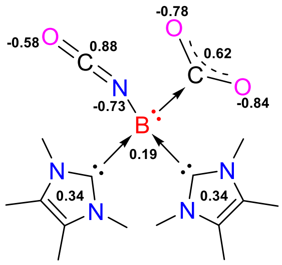

Supplementary Figure 36. Grouped NPA charges of compounds **1** and **3** at M06-2X/def2-TZVP level.

**Compound**	NPA data
**2**	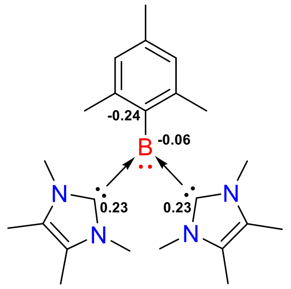

Supplementary Figure 37. NPA charges of compound **2** at M06-2X/def2-TZVP level.

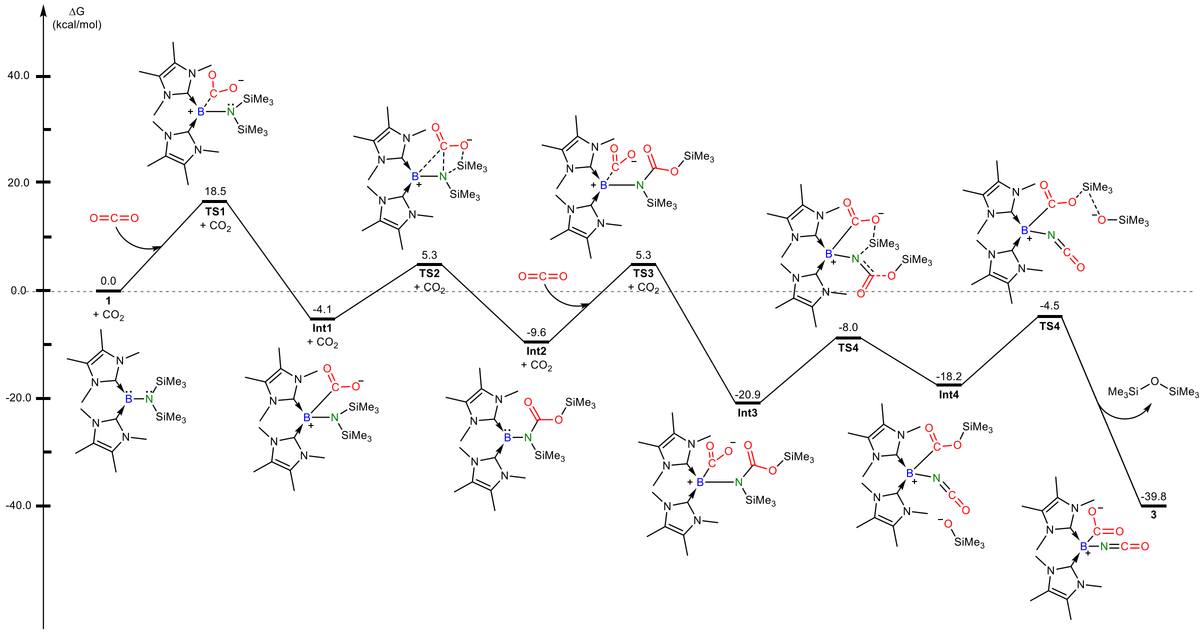

Supplementary Figure 38. DFT-calculated free energy profile (kcal·mol^{-1}) using IEFPCM solvation model at M06-2X/def2-TZVP level for the proposed mechanism of the conversion of **1** to **3**.

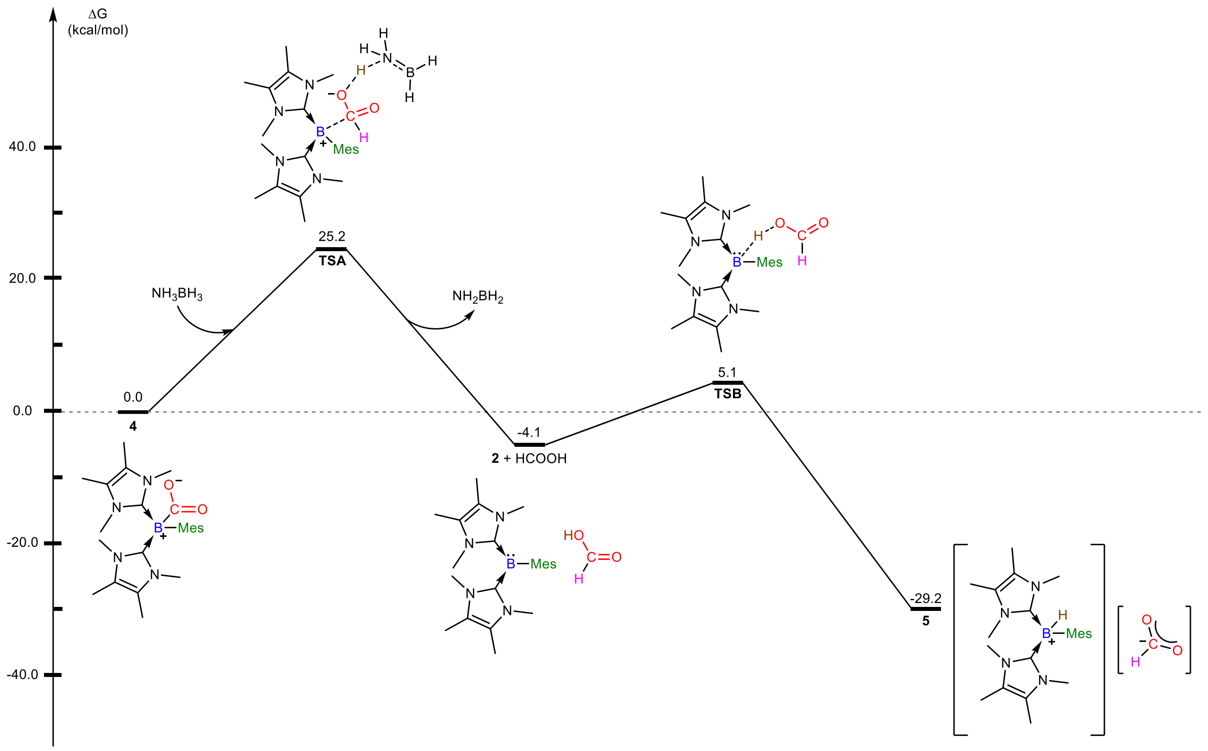

Supplementary Figure 39. DFT-calculated free energy profile (kcal·mol^{-1}) using IEFPCM solvation model at M06-2X/def2-TZVP level for the proposed mechanism of the conversion of **4** to **5**.

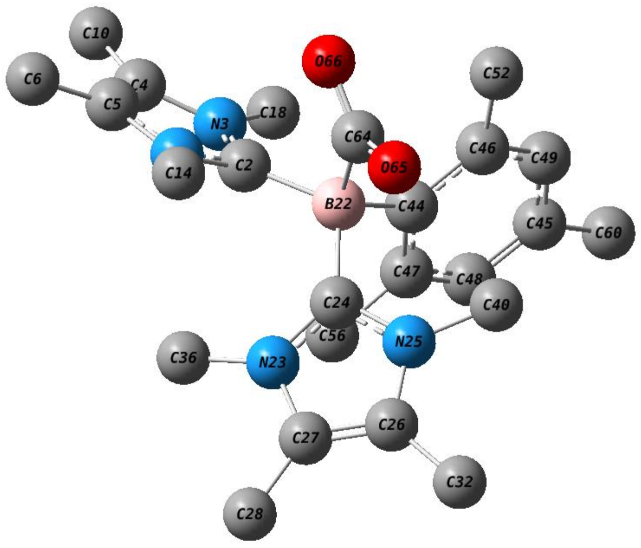

Supplementary Figure 40. Optimized structure of compound **4**. Hydrogen atoms are omitted for clarity. Selected bond lengths (Å) and angles (deg): B22-C2 1.631, B22-C24 1.637, B22-C44 1.649, C22-C64 1.705, C64-O65 1.250, C64-O66 1.253, C2-B22-C24 114.8, C24-B22-C44 105.3, C2-B22-C44 114.7, C2-B22-C64 98.0, C24-B22-C64 106.4, C44-B22-C64 117.7, O65-C64-O66 126.7.

3. Supplementary References

[1] Neilson, R. H., Li, B. L., Goodman, M. A. Synthesis and stereochemistry of some alkyl[bis(trimethylsilyl)amino]boranes. *Inorg. Chem.* **23**, 1368-1371 (1983).

[2] Jäkle, F., Sundararaman. A. A comparative study of base-free arylcopper reagents for the transfer of aryl groups to boron halides. *J. Organomet. Chem.* **681**, 134-142 (2003).

[3] Sheldrick, G. M. SADABS V2014/4 (Bruker AXS Inc.), University of Göttingen, Germany, 2014.

[4] Sheldrick, G. M. SHELXL-2014/6 (Sheldrick, 2014); Bruker AXS Inc., Madison, WI, USA, 2014.

[5] Zhao, Y.; Truhlar, D. G. The M06 suite of density functionals for main group thermochemistry, thermochemical kinetics, noncovalent interactions, excited states, and transition elements: two new functionals and systematic testing of four M06-class functionals and 12 other functionals. *Theor. Chem. Acc.* **120**, 215-241 (2008).

[6] Weigend, F.; Ahlrichs, R. Balanced Basis Sets of Split Valence, Triple Zeta Valence and Quadruple Zeta Valence Quality for H to Rn: Design and Assessment of Accuracy. *Phys. Chem. Chem. Phys.* **7**, 3297−3305 (2005).

[7] Frisch, M. J., Trucks, G. W., Schlegel, H. B., Scuseria, G. E., Robb, M. A., Cheeseman, J. R., Scalmani, G., Barone, V., Mennucci, B., Petersson, G. A., Nakatsuji, H., Caricato, M.; Li, X., Hratchian, H. P., Izmaylov, A. F., Bloino, J., Zheng, G., Sonnenberg, J. L., Hada, M., Ehara, M., Toyota, K., Fukuda, R., Hasegawa, J., Ishida, M., Nakajima, T., Honda, Y., Kitao, O., Nakai, H., Vreven, T., Montgomery, Jr., J. A., Peralta, J. E., Ogliaro, F., Bearpark, M., Heyd, J. J., Brothers, E., Kudin, K. N., Staroverov, V. N., Keith, T., Kobayashi, R., Normand, J., Raghavachari, K., Rendell, A., Burant, J. C., Iyengar, S. S., Tomasi, J., Cossi, M., Rega, N., Millam, J. M., Klene, M., Knox, J. E., Cross, J. B., Bakken, V., Adamo, C., Jaramillo, J., Gomperts, R.; Stratmann, R. E., Yazyev, O., Austin, A. J., Cammi, R., Pomelli, C., Ochterski, J. W., Martin, R. L., Morokuma, K., Zakrzewski, V. G., Voth, G. A., Salvador, P., Dannenberg, J. J., Dapprich, S., Daniels, A. D., Farkas, O., Foresman, J. B., Ortiz, J. V.; Cioslowski, Martin, R. L., K., Morokuma; Ö ., Farkas; J. B., Foresman; D. J., Fox, Gaussian 16; revision C.01; Gaussian, Inc.: Wallingford CT, 2016.

[8] Cance`s, M. T.; Mennucci, B.; Tomasi, J. A new integral equation formalism for the polarizable continuum model: Theoretical background and applications to isotropic and anisotropic dielectrics. *J. Chem. Phys.* **107**, 3032-3041 (1997).

[9] Cossi, M.; Barone, V.; Mennucci, B.; Tomasi, J. Ab initio study of ionic solutions by a polarizable continuum dielectric model. *J. Chem. Phys. Lett.* **286**, 253-260 (1998).

[10] Mennucci, B.; Tomasi, J. Continuum solvation models: A new approach to the problem of solute's charge distribution and cavity boundaries. *J. Chem. Phys.* **106**, 5151-5158 (1997).
